# Investigation of Thiazolidine‐2,4‐Dione Derivatives as Acetylcholinesterase Inhibitors: Synthesis, *In Vitro* Biological Activities and *In Silico* Studies

**DOI:** 10.1002/open.202400294

**Published:** 2025-01-10

**Authors:** Hanane Naeimi, Maryam Taheri, Hossein Ghafouri, Asadollah Mohammadi

**Affiliations:** ^1^ Department of Biology Faculty of Basic Sciences University of Guilan Rasht 4193833697 Iran; ^2^ Department of Chemistry Faculty of Sciences University of Guilan Rasht 4193833697 Iran

**Keywords:** Alzheimer's disease (AD), Acetylcholinesterase (AChE), Thiazolidine-2,4-dione (TZD), Biological activity, Inhibitors

## Abstract

The inhibition of acetylcholinesterase (AChE), an enzyme responsible for the inactivation and decrease in acetylcholine in the cholinergic pathway, has been considered an attractive target for small‐molecule drug discovery in Alzheimer's disease (AD) therapy. In the present study, a series of TZD derivatives were designed, synthesized, and studied for drug likeness, blood–brain barrier (BBB) permeability, and adsorption, distribution, metabolism, excretion, and toxicity (ADMET). Additionally, docking studies of the designed compounds were performed on AChE. Additionally, all the TZD derivatives (CHT1‐5) showed an acceptable affinity for AChE inhibition, and the results showed convincing binding modes in the active site of AChE. Among them, 5‐(4‐methoxybenzylidene) thiazolidine‐2,4‐dione (CHT1) was identified as the most potent AChE inhibitor (IC_50_ of 165.93 nM) with the highest antioxidant activity. Following the exposure of PC12 cells to Aβ1‐42 (100 μM), a marked reduction in cell survival was observed. Pretreatment of PC12 cells with TZD derivatives had a neuroprotective effect and significantly enhanced cell survival in response to Aβ‐induced toxicity. Western blotting analysis revealed that CHT1 (5 and 8 μM) downregulated p‐Tau and HSP70 expression levels. The results indicate that CHT1 is a promising and effective AchE‐I that could be utilized as a powerful candidate against AD.

## Graphical abstract

Thiazolidin‐2,4‐dion derivatives were synthesized and evaluated for drug‐likeness, BBB permeability, and ADMET properties. Docking studies revealed that all derivatives (CHT1‐5) had good AChE inhibition, although CHT1 was the most effective and showed strong antioxidant activity *in vitro*. CHT1 pre‐treatment enhanced PC12 cell survival against Aβ toxicity, and Western blotting indicated decreased p‐Tau and HSP70 levels.

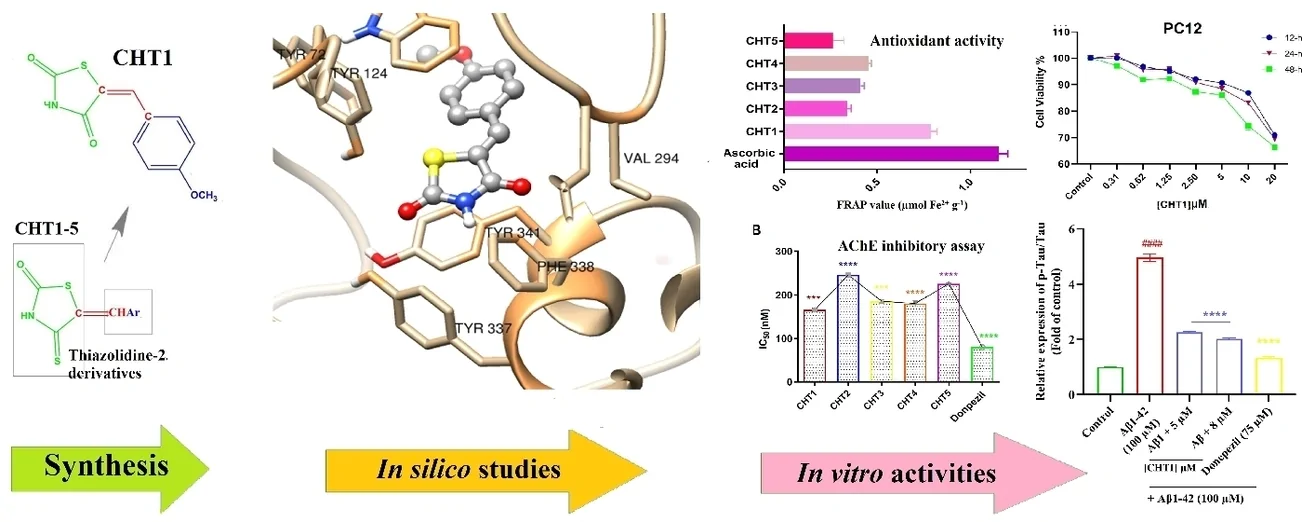

## 1 Introduction

The global population of elderly people is on the rise, which results in an increase in the incidence of chronic degenerative diseases, central nervous system damage, and dementia, such as Alzheimer's disease (AD).^[1]^ The brain is involved in AD, a devastating condition that causes a decrease in cognitive ability. AD is a progressive disorder that can cause irreversible damage to memory, language, and thinking ability.^[2]^ Two main cholinesterases (ChEs), acetylcholinesterase (EC 3.1.1.7; AChE) and butyrylcholinesterase (BChE; EC 3.1.1.8), function in the cholinergic system and are critical enzymes for this pathway.^[3]^ By breaking down acetylcholine at the neural synapse, AChE is responsible for terminating neurotransmitters. A decrease in acetylcholine concentrations causes increased inflammation, apoptosis, and oxidative stress in age‐related neurodegenerative disorders, such as dementia and AD.^[4]^ Evidence has also shown that AChE may be linked to the appearance of senile plaques in the brain, which can lead to neurodegenerative disorders such as Alzheimer's disease.^[5]^ Research suggests that the assembly of senile plaques of beta‐amyloid (Aβ) and neurofibrillary tangles (NFTs) of p‐Tau in the brain can cause neurodegenerative disorders such as Alzheimer's disease. Beta‐amyloid peptide plays a crucial role in AD.^[6]^

Neurons express a high amount of the microtubule‐associated protein called tau. Tau is an inherently destructive protein that cooperates with microtubules by mutation or posttranslational modifications, such as phosphorylation, in disorders such as AD.^[7]^ Tau tends to aggregate and form NFTs. These events eventually lead to neuronal cell death and neurodegenerative diseases such as dementia and AD.^[8]^ Additionally, oxidative stress is an influential pathologic factor in neurodegenerative illnesses that contributes to the progression of disease. Neuronal damage can result from protein aggregation and reactive oxygen species generation.^[9]^

Furthermore, heat shock proteins (HSPs) are a vital class of molecular chaperones that are highly expressed in stress conditions such as hypoxia, oxidative stress, and various diseases, such as cancer, AD, and inflammatory disease.^[10]^ However, the negative effect of the upregulation of these proteins can be exacerbated by chronic inflammation.^[11]^ Tau homeostasis is typically managed by molecular chaperones, such as HSP70.^[12]^ HSP70 is strongly linked to tau accumulation, and in the central nervous system (CNS), HSP70 seemingly plays an influential protective role.^[13]^ Numerous inhibitors (such as rhodacyanine and MKT‐077) against HSP70 have been identified, resulting in the degradation of tau protein in cells, neurons, and brain tissue.^[14]^ Our previous study and several other studies revealed that by reducing the expression level of HSP70, the expression level of tau decreased.^14^, ^15^ Nonetheless, the exact basis of the mechanism of action of HSP70 is not clear.

There are sufficient reports of cholinergic dysfunction in the AD brain. The clinical treatment for AD involves restoring the cholinergic system after injury.^[16]^ The main classes of AChE inhibitors (AchE‐Is) include galanthamine, donepezil, rivastigmine, and tacrine, which are FDA‐approved therapeutic approaches for managing AD.^[17]^

To date, many compounds based on thiazol base have been identified as potent cholesterol esterase, AChE,^[18]^ and BChE inhibitors in the AD therapy pathway.^[19]^ Thiazolidine‐2,4‐dione (TZD) derivatives have been applicable for their ability to target various diseases, such as diabetes, viruses, inflammation, seizures, infections, heart disease, arthritis, and parasites.^[20]^ Because of their expansive pharmacological specificity, TZDs are currently being investigated as better, safer, and potential pharmacological agents.^[21]^ Comprehensive studies on TZD derivatives have led to registering 824 patents (https://pubchem.ncbi.nlm.nih.gov/). In previous studies, we demonstrated the anticholinesterase effects of TZD derivatives on an AD rat model,^[15b,d]^ and in another study, the TZD acts as an M1 muscarinic acetylcholine receptor agonist, enhanced the expression of choline acetyltransferase (ChAT). Consequently, these compounds improved the symptoms of AD both *in vitro* and *in vivo* models.^[22]^ The current investigation focused on synthesizing a different series of derivatives of TZD and evaluating its *in vitro* biological activity and inhibitory effects on AChE. Our team also explored *in silico* insights through structural studies of the synthesized compounds using molecular docking and ADMET studies.

## Experimental and Computational Methods

### Materials

Chemical materials for synthesis, enzyme assays, and amyloid beta 1–42 (Aβ1‐42) were obtained from Sigma–Aldrich, while chemical solutions were sourced from Merck. The following chemicals and kits were acquired from respective suppliers: mouse anti‐rabbit IgG‐HRP (sc‐2357), m‐IgGκBP‐HRP (sc‐516102), β‐Actin (C4) (sc‐47778), HSP70 (C92F3A‐5) (sc‐66048) from Santa Cruz Biotechnology, Inc., and phospho‐Tau (Ser404) (D2Z4G) from Cell Signaling Technology.

### Chemistry

#### General Experimental Methods for the Synthesis of TZD‐Based Derivatives

In a 50 mL round‐bottom flask equipped with a magnetic stirrer, thiazolidinone (1 mmol) and aromatic aldehyde (1 mmol) were dissolved in 15 mL of ethanol, and 2–3 drops of piperidine were added as an organic catalyst to the solution and refluxed for 9–10 hours. The reaction progress was checked by thin‐layer chromatography (TLC) with a mixture of n‐hexane/ethyl acetate. After completion, the temperature reached ambient temperature, the solution was neutralized with acetic acid, and the precipitate was filtered using Whatman® cellulose filter paper. The final compounds were clarified by recrystallization after being placed in an oven at 40 °C for 24 hours. The melting points were recorded using a Mettle IA9000 apparatus. Additionally, IR spectra were recorded using a Shimadzu FT‐IR 4100 spectrophotometer in KBr pellets. Recording ^1^H and ^13^C NMR spectra of the synthesized compounds was recorded using an FT‐NMR (400 MHz) Brucker apparatus. The chemical shifts in ppm were determined using TMS as an internal standard, and coupling constants (J) were calculated in Hz. Furthermore, an electrothermal melting point device was used to determine the melting points of the synthesized compounds (mp).

##### 5‐(4‐Methoxybenzylidene)thiazolidine‐2,4‐dione (CHT1)

Yellow solid (yield 95 %); mp=202–204 °C; FT‐IR (KBr) ν cm^−1^: 3423.97 (NH), 2959.80 (C−H), 2929.21 (C−H), 1728.77 (C=O), 1284.04 (C−O), 1126.88, 1072.85. ^1^H NMR (400 MHz, DMSO‐d_6_, Me_4_Si) δ_H_ ppm: 3.84 (3 H, s, OMe), 7.11 (2 H, d, J=8.80 Hz, H_3_ & H_5_ of 4‐OMeC_6_H_4_), 7.57 (2 H, d, J=8.80 Hz, H_2_ & H_6_ of 4‐OMeC_6_H_4_), 7.76 (1 H, s, C=CH), 12.53 (1 H, br s, NH). ^13^C NMR (100 MHz, DMSO‐d_6_) δ_C_ ppm: 55.96, 115.39 (2 C), 120.73, 125.96, 132.33 (2 C), 132.58, 161.46, 167.93, 168.46.

##### 5‐(4‐Chlorobenzylidene)thiazolidine‐2,4‐dione (CHT2)

Yellow solid (yield 94 %); mp=210–214 °C; FT‐IR (KBr) ν cm^1^: 3146.32 (NH), 1723.42 (C=O), 1608.48, 1167.03, 815.62, 697.46, 516.44. ^1^H NMR (400 MHz, DMSO‐d_6_, Me_4_Si) δ_H_ ppm: 7.61 (4 H, m, H_2_, H_3_, H_5_ & H_6_ of 4‐ClC_6_H_4_), 7.79 (1 H, s, C=CH), 12.69 (1 H, br s, NH). ^13^C NMR (100 MHz, DMSO‐d_6_) δ_C_ ppm: 124.83 (2 C), 129.87 (2 C), 130.89, 132.11, 132.44, 135.47, 167.75, 168.14.

##### 5‐(3‐Hydroxybenzylidene)thiazolidine‐2,4‐dione (CHT3)

Yellow solid (yield 90 %); mp=260–263 °C; FT‐IR (KBr) ν cm^−1^: 3302.32 (NH), 3174.97(OH), 1752.01 (C=O), 1691.67 (C=O), 1591.14 (C=C), 1447.72, 1304.53, 1144.94, 679.98. ^1^H NMR (400 MHz, DMSO‐d_6_, Me_4_Si) δ_H_ ppm: 6.90 (1H, d, J=8.00 Hz, H_4_ of 3‐OHC_6_H_4_), 6.99 (1 H, s, H_2_ of 3‐OHC_6_H_4_), 7.04 (1 H, d, J=7.60 Hz, H_6_ of 3‐OHC_6_H_4_), 7.34 (1 H, t, J=8.00 Hz, H_5_ of 3‐OHC_6_H_4_), 7.70 (1 H, s, C=CH), 9.89 (1 H, s, OH), 12.62 (1 H, br s, NH). ^13^C NMR (100 MHz, DMSO‐d_6_) δ_C_ ppm: 116.39, 118.22, 121.81, 123.76, 130.87, 132.48, 134.66, 158.34, 167.83, 168.47.

##### 5‐(2‐Hydroxybenzylidene)thiazolidine‐2,4‐dione (CHT4)

Yellow solid (yield 94 %); mp=254–259 °C; FT‐IR (KBr) ν cm^−1^: 3415.95 (NH), 3132.71 (OH), 3014.33 (C−H), 1729.26 (C=O), 1674.24 (C=O), 1590.44 (C=C), 1454.48, 1333.64, 1247.99, 1194.17, 1163.13, 688.38, 560.75. ^1^H NMR (400 MHz, DMSO‐d_6_, Me_4_Si) δ_H_ ppm: 6.94 (1 H, d, J=7.60 Hz, H_3_ of 2‐OHC_6_H_4_), 6.97 (1 H, t, J=8.80 Hz, H_5_ of 2‐OHC_6_H_4_), 7.33 (2 H, m, H_4_ & H_6_ of 2‐OHC_6_H_4_), 8.05 (1 H, s, C=CH), 10.4 (1 H, s, OH), 12.53 (1 H, br s, NH). ^13^C NMR (100 MHz, DMSO‐d_6_) δ_C_ ppm: 116.60, 120.14, 120.43, 122.34, 127.50, 128.77, 132.71, 157.78, 167.99, 168.65.

##### 5‐((Naphthalene‐1‐yl)methylene)thiazolidine‐2,4‐dione (CHT5)

Yellow solid (yield 93 %); mp=141–143 °C; FT‐IR (KBr) ν cm^−1^: 3422.41(NH), 2960.09 (C−H), 1738.19 (C=O), 1686 (C=O), 1564.38 (C−C), 1330.64, 1147.38, 769.84, 677.66, 549.91. ^1^H NMR (400 MHz, DMSO‐d_6_, Me_4_Si) δ_H_ ppm: 7.65 (4 H, m, H_3_, H_4_, H_5_ & H_8_ of 2‐naphtyl), 8.06 (1 H, d, J=7.60 Hz, H_2_ of 2‐naphtyl), 8.09 (1 H, d, J=8.40 Hz, H_6_ of 2‐naphtyl), 8.15 (1 H, d, J=8.50 Hz, H_7_ of 2‐naphtyl), 8.44 (1 H, s, C=C−H), 12.71 (1 H, br s, NH). ^13^C NMR (100 MHz, DMSO‐d_6_) δ_C_ ppm: 123.88, 126.11, 126.80, 127.31, 127.84, 127.94, 128.90, 129.37, 130.82, 131.19, 131.46, 133.76, 167.70, 168.81.

### In Vitro Studies

#### FRAP Radical Scavenging Activity

The ferric reducing/antioxidant power (FRAP) assay was performed based on a previous study.^[15a]^ The principle required reducing ferric 2,4,6‐tris(2‐pyridyl)‐1,3,5‐triazine (Fe^3+^‐TPTZ) to iron while TZD derivatives (CHT1‐5) were present. A standard curve was drawn to analyze different concentrations of ferrous sulfate (0.1–1 mM). The analysis was conducted in triplicates and the value of antioxidant activity results were expressed in mmol ferrous ion equivalents per gram of test compounds (mmol Fe^2+/^g).

#### ChEs Inhibition Assay

The ChEs inhibitory activities based on Ellman's method were assessed using previously described methods.^[15b]^ Acetylcholinesterase (*electric eel, Torpedo californica*) inhibition assays were conducted with ATCI (10 mM) and DTNB (10 mM). The experiment required adding 50 μL of AChE in Tris‐HCl buffer (pH 8.0), 100 μL substrate, and 50 μL of the sample dissolved in DMSO (10 % in Tris‐HCl buffer) to the wells. For BChE inhibition, mixtures contained 30 μL of 10 mM DTNB, 5 μL of BChE, 30 μL of 10 mM BTChCl (10 mM), and 10 μL of samples.

After incubating the plate at 37 °C for 30 minutes the absorbance was measured at 405 nm for 60 minutes in a Bioteck microplate reader. As a standard drug, donepezil was dissolved in 10 % DMSO in Tris‐HCl buffer at a concentration of 1 mg/mL. All samples were dissolved in 10 % DMSO, and the experiment was performed in triplicate.

#### Cell Culture and MTT Assay

*Rattus norvegicus* pheochromocytoma PC12 cells (Pastor Institute, Iran) were cultured in Dulbecco's modified Eagle's medium (DMEM) supplemented with 10 % fetal bovine serum (FBS) and 1 % penicillin/streptomycin (Gibco) at 37 °C with 5 % CO_2_. PC12 cells were treated with different concentrations of CHT1 for 12, 24, or 48 hours, and promising results were obtained.

In addition, the Aβ1‐42 peptides were diluted in water and preaggregated at 37 °C for 7 days before use. The cellular AD model was successfully constructed *in vitro* by incubating PC12 cells with 100 μM aggregated Aβ for 24 hours. DMSO (50 μL) and 0.5 mg/mL MTT (10 μL) stock solution were added to each well for 4 hours of incubation. After 30 minutes, the plates were shaken, and the optical density was measured at 570 nm using an ELISA reader (Biotech). The MTT assay used by Pirali et al.,^[23]^ which underwent slight modifications, yielded promising results.

#### Western Blot Analysis

PC12 cells were seeded in a 6‐well plate, treated CHT1 at concentrations of 5 and 8 μM for 24 hours, washed with cold PBS, and lysed with lysis buffer for 30 minutes on ice. After centrifugation at 12,000×g for 10 minutes at 4 °C, the lysates were transferred to new tubes. The Bradford assay was utilized to determine the protein concentration.^[24]^ SDS–PAGE was used to separate the denatured proteins, which were subsequently transferred to PVDF membranes. The membranes were blocked with nonfat dry milk in PBS containing 0.1 % Tween‐20 (PBST) and then incubated overnight at 4 °C with primary antibodies against p‐Taul/Tau, HSP70, and β‐actin as a control. Subsequently, the membranes were exposed to a secondary antibody conjugated with horseradish peroxidase (HRP) for 2 hours at room temperature. Using the densitometric scanning technique in the ImageJ program, the density of the protein bands in the relative direction was determined.

### In Silico Studies

#### BBB Prediction, ADME, and Toxicity

BBB penetration is a principal parameter affecting biological activity. Drugs that target the CNS, such as AchE‐Is, must cross the BBB. The Online BBB Predictor^[25]^ was utilized to determine the capability of the synthesized compounds.

The ADME features of synthesized TZD derivatives were calculated and estimated by the free admetSAR server. The server is based on cheminformatics and employs 50 high‐quality QSAR models to predict the ADMET properties of chemicals. This server has more than 200,000 data points and 96,000 unique combinations. The 30 models of the admetSAR tool (http://lmmd.ecust.edu.cn/admetsar2) prediction for new chemical properties and ADMET *in silico* filters were constructed.^[26]^

Lipinski's ′Rule of Five’ (drug likeness) was calculated using the MProTox‐II online property estimation toolkit (available at https://tox‐new.charite.de/protox_II/), which defines the standards for drug likeness properties.^[27]^

#### Molecular Docking Process

The interactions between TZD derivatives and AChE were investigated by conducting docking studies using AutoDock 4.2, which utilizes the Lamarckian Genetic Algorithm (LGA).^[28]^ For this purpose, the crystallographic structure of human acetylcholinesterase (PDB ID: 4 M0E) was obtained from the Protein Data Bank (RCSB). A series of steps were carried out to prepare the enzyme and compounds for docking studies. The protein was prepared by removing water, cofactor, and co‐crystallized ligands but polar and nonpolar hydrogens were added using ICM‐Pro (Molsoft, L.L.C.) (http://www.molsoft.com/). The structure of the ligands was generated using ChemDraw (http://www.chemaxon.com) and transformed into a 3D format using ICM‐Pro. Finally, the pdbqt file was generated after adding the Kollman and Gasteiger charges to the protein and polar hydrogens to the ligands for further analysis.^[28]^ Whole chain A was considered a grid box with a size of 126×126×126 Å, and the center of the grid box was located at x=−3.005, y=−40.058, and z=31.052. All docking calculations treated the protein as rigid and the ligand as flexible. The free binding energies of all compounds were calculated, and the lowest free binding energy was selected for further analysis.

### Statistical Analysis

GraphPad Prism 8 (GraphPad, La Jolla, CA, USA) was used for all the statistical analyses. In three independent experiments, the concentrations were tested in triplicate (n=3). The mean±SD values were used to determine significant differences from controls using one‐way ANOVA. When the *P* value was less than 0.05, the statistical significance of differences was determined. Two‐tailed t tests were used to compare groups.

## 2 Results and Discussion

### 2.1 Chemical Characterization

**Scheme 1:**
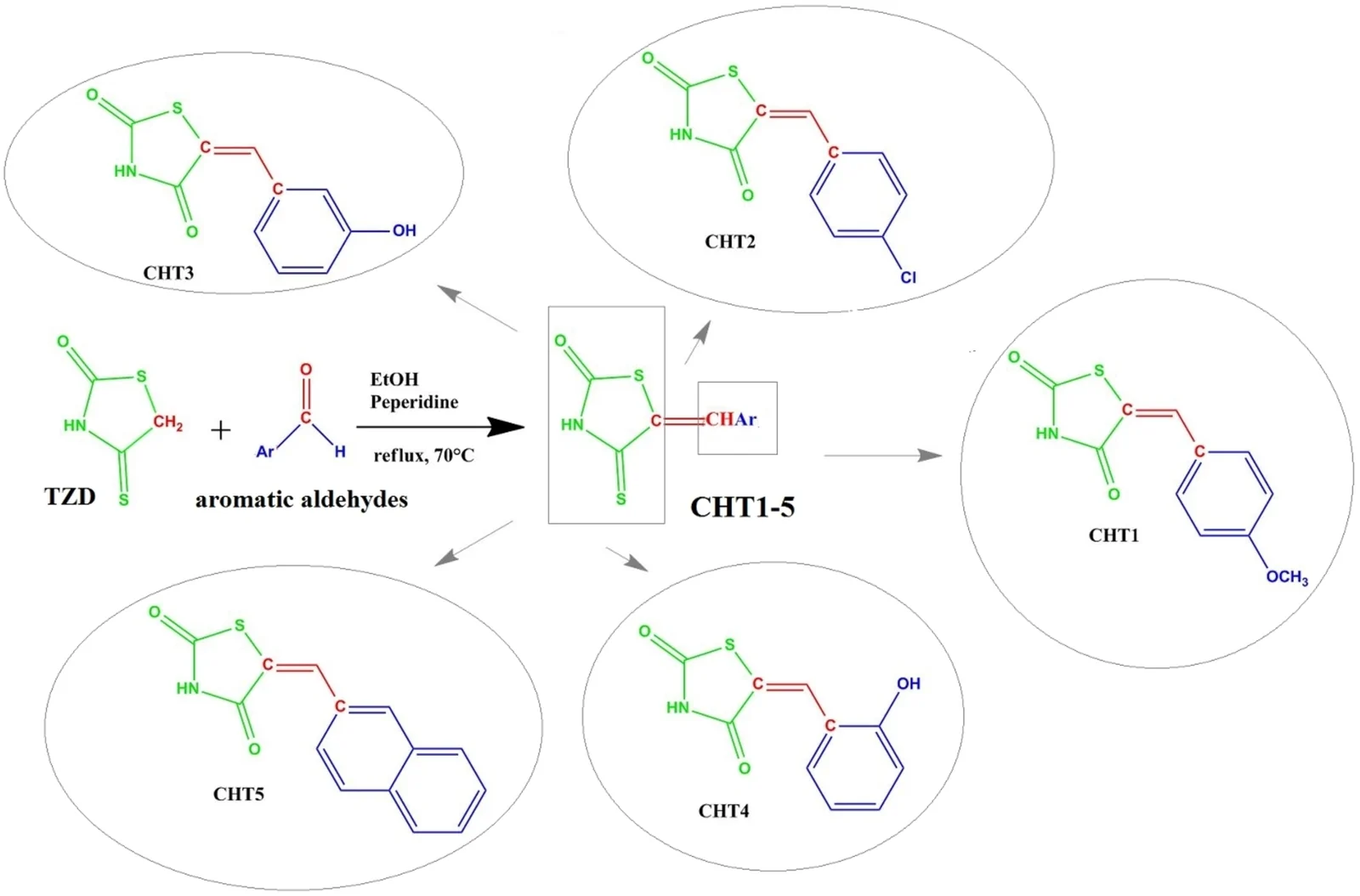
Synthesis pathway of the thiazolidine‐2,4‐dione derivatives (CHT1‐CHT5). TZD and aromatic aldehydes were dissolved in ethanol, and 2–3 drops of piperidine (as a catalyst) were added and refluxed for 9–10 h to obtain TZD derivatives (CHT1‐CHT5).

Designing, synthesizing, and delivering molecules that can be beneficial for human therapeutic purposes is crucial in the fields of medicinal chemistry and medicine. One such molecule is thiazolidines, which have diverse potential for biological activity. The present study involved designing, synthesizing, and assessing various TZD derivatives for which their thiazol base was efficacious.^[29]^ Thiazolidinediones, or glitazones, are a drug class explored for neurodegenerative disorders. Pioglitazone and rosiglitazone, antidiabetic TZD drugs, have been studied in clinical trials for their potential to combat cognitive decline linked to AD.^[30]^ Thiazole rings, five‐membered heterocycles containing nitrogen and sulfur, have been extensively studied in AD research. Their diverse biological activities make them valuable in drug development, with over 90 thiazole‐containing derivatives currently in clinical trials.^[31]^ The thiazolidine‐2,4‐dione and formyl derivatives were used to synthesize Schiff bases. The TZD‐based Schiff bases were prepared through the condensation reaction of thiazolidine‐2,4‐dione with various aromatic aldehyde derivatives in ethanol. Scheme 1 shows the synthesis pathway of TZD derivatives. The E‐configuration of synthesized compounds is more rational because it experiences less steric hindrance, leading to increased stability. The complete characterization of all compounds was achieved through detailed spectral studies using FT‐IR, ^1^H NMR, and ^13^C NMR spectroscopic data. To obtain spectroscopic data, refer to Figures S1–S15, in Supplementary material.

### 2.2 Biological Activity

#### 2.2.1 Effects of CHT1‐5 on FRAP Radical‐Scavenging

**Figure 1:**
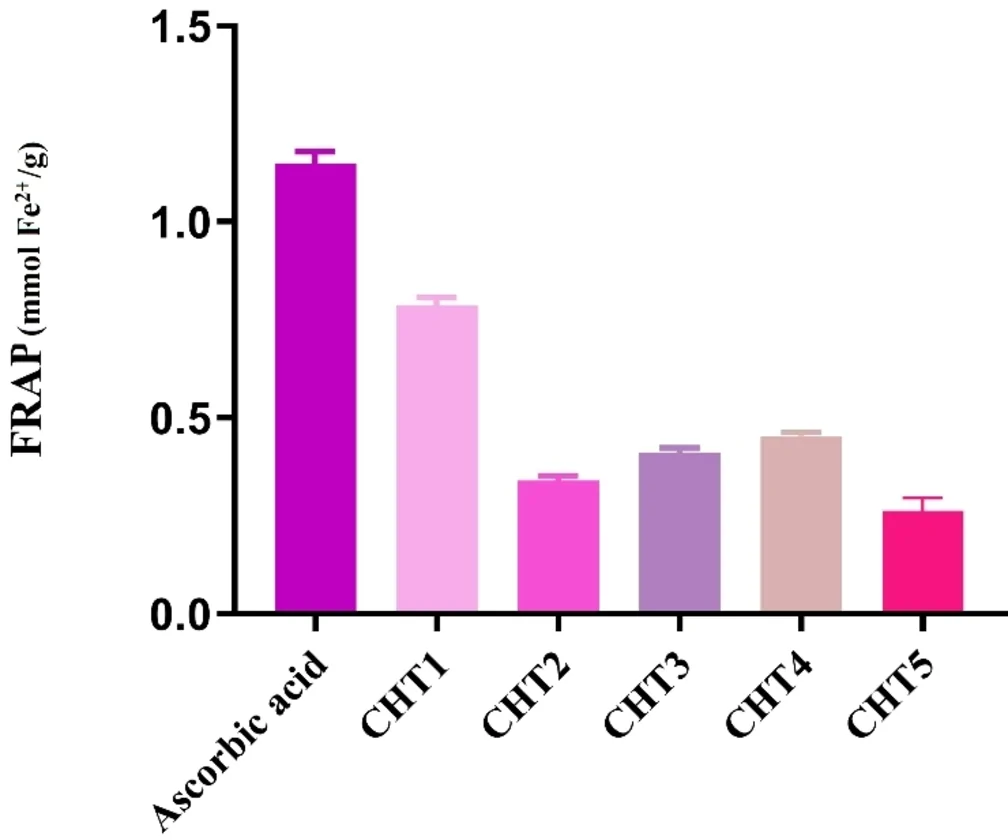
Antioxidant activities (mmol/g) of CHT1‐5. The scavenging activity of CHT1‐5 at different concentrations of Fe^2+^ indicates significant antioxidant activity of CHT derivatives. Ascorbic acid was used as a standard.

The antioxidant capacity of TZD derivatives CHT1‐5 was assessed using a ferric reducing/antioxidant power (FRAP) assay. Ascorbic acid was used as a positive control. The antioxidant capacities of the rest compounds were calculated as follows: ascorbic acid (1.15 mmol Fe^2+^/g)>CHT1 (0.78 mmol Fe^2+^/g)>CHT4 (0.45 mmol Fe^2+^/g)>CHT3 (0.41 mmol Fe^2+^/g)>CHT2 (0.33 mmol Fe^2+^/g)>CHT5 (0.26 mmol Fe^2+^/g). The results in Figure 1 indicate that CHT1 has more powerful antioxidant activity than other derivatives due to its methoxy group. Experimental studies indicate that methoxy substitutions influence the scavenging rate of reactive oxygen species variably based on their position. Specifically, para substitution enhances activity due to the mesomeric effect, while meta substituents have no impact on activity.^[32]^ Chen et al.,^[33]^ and Jeong et al.,^[34]^ noted that the methoxyl (−OCH_3_) group can enhance the antioxidant activities of phenolic acids. However, the other compounds did not display considerable antioxidant activity.

#### 2.2.2 Effects of CHT1‐5 on ChEs Activity

**Figure 2:**
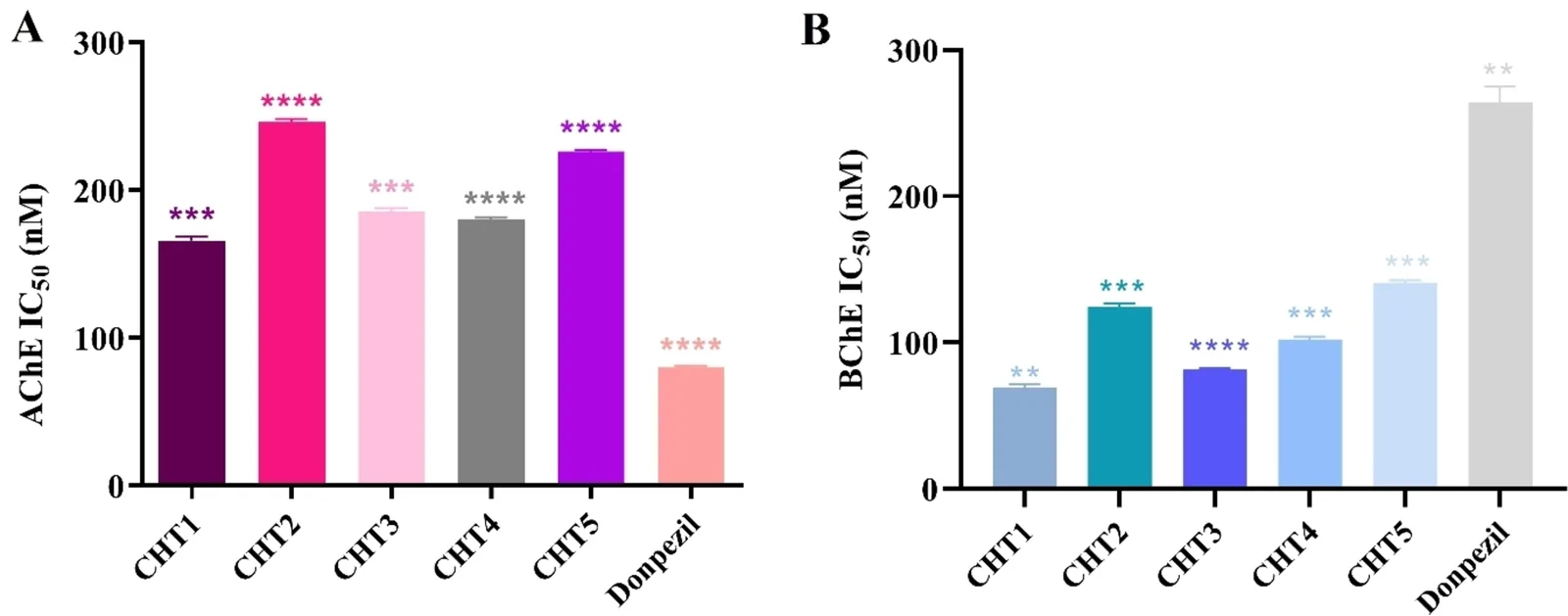
The IC_50_ values (nM) of CHT1‐5 associated with AChE activity. The *in vitro* inhibitory concentration (IC_50_) of CHT derivatives toward AChE (A) and BChE (B) are shown. Donepezil was utilized as a positive control. The values are presented as the means±SDs (n=3). *P* values <0.05 were considered to indicate statistical significance. *p <0.05, **p <0.01, ***p <0.001, ****p <0.0001 indicate significant differences according to two‐tailed t tests.

The *in vitro* inhibitory activities against AChE and BChE were assessed against donepezil, a standard drug, for all synthesized compounds CHT1‐5.^[15a]^ The AChE IC_50_ values were measured for different TZD derivatives: donepezil (79.99 nM)>CHT1 (165.93 nM)>CHT4 (180.36 nM)>CHT3 (185.68 nM)>CHT5 (225.96 nM)>CHT2 (245.80 nM). The BChE IC_50_ values for these compounds are as follows: CHT1 (69.07 nM)>CHT3 (81.88 nM)>CHT4 (102.24 nM)>CHT2 (124.56 nM)>CHT5 (140.77 nM)>donepezil (264.23 nM). According to the IC_50_ values shown in Figure 2A, B, CHT1 with a methoxy group appears to have the highest AChE and BChE inhibitory activity among all the derivatives. The results indicate that these compounds selectively target BChE over AChE. Donepezil significantly inhibits AChE with high selectivity over BChE.^[35]^ The data also confirmed this. This indicates that AChE inhibition is a key therapeutic target in AD treatment strategies.^[15d]^ CHT1, which contains a methoxy group at the para position of the phenyl ring, showed the most potent inhibitory effect on AChE and BChE. Compounds containing methoxy groups demonstrate significant AChE inhibition. The methoxy group enhances the inhibitory activity of the compound against AChE, especially when located at specific sites on the molecule.^[36]^ Çi̇ftci̇ and et al indicated that Methoxy groups at both positions strongly inhibit AChE, while one methoxy group shows minimal effect, underscoring their importance for inhibition.^[36b]^ According to this information, the chemical structure of a compound is a significant factor in its inhibitory activity. Further research could investigate the potential of CHT1 in treating conditions linked to AChE dysfunction in neurodegenerative disorders. CHT1 was selected for additional cell studies due to the antioxidant activity and AChE inhibitory effects of TZD derivatives

#### 2.2.3 Effects of CHT1 on Aβ‐Induced Cell Viability and Toxicity

**Figure 3:**
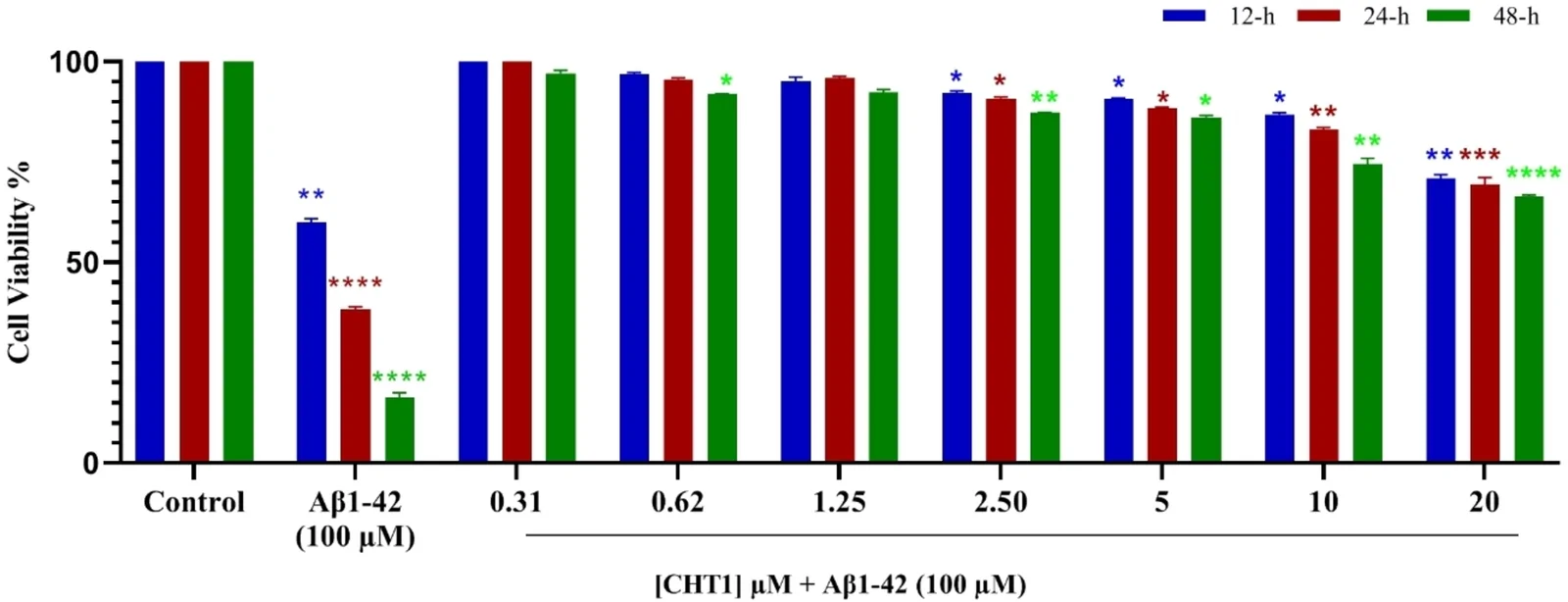
The protective effects of CHT1 at different concentrations against Aβ1‐42. Cytotoxicity in PC12 neuronal cells can be caused by Aβ1‐42 (100 μM). CHT1 pretreatment led to a significant reduction in Aβ1–42‐induced cell death in a dose‐dependent manner. These changes are shown in the bar graphs. The values are presented as the means±SDs (n=3). *P* values <0.05 were considered to indicate statistical significance. *p <0.05, **p <0.01, ***p <0.001 ****p <0.0001 represent a significant difference according to one‐way ANOVA.

The MTT assay was utilized to evaluate the protective impact of CHT1 against Aβ1‐42‐induced toxicity in PC12 cells (0.31–20 μM CHT1, 12, 24, and 48 h). CHT1 pretreatment led to a significant reduction in Aβ1‐42‐induced cell death in a dose‐dependent manner, and acute toxicity was not observed at the defined doses (Figure 3). The cell viability of the control groups that were not exposed to CHT1 or Aβ1‐42 was defined as 100 %. The cells exposed to Aβ1‐42 showed a decrease in viability from 12 h to 48 h (59.9–16.4 %). The findings indicated that the nontoxic concentration of CHT1 was 20 μM, and its viability percentage was 66.5 % of that of the control after 48 h. These findings further support the probable therapeutic potential of CHT1 in protecting neuronal cells against Aβ1‐42 toxicity and its noncytotoxic effect on cell viability according to the MTT assay. The methoxy substituent based on thiazol‐based compounds plays a significant role in drug design by enhancing biological activity and pharmacological properties. This makes methoxy groups valuable in optimizing drug interactions and improving performance.^20^, ^37^

#### 2.2.4 Effect of CHT1 on Aβ‐Induced p‐Tau, Tau, and HSP70 Expression Levels in PC12 Cells

**Figure 4:**
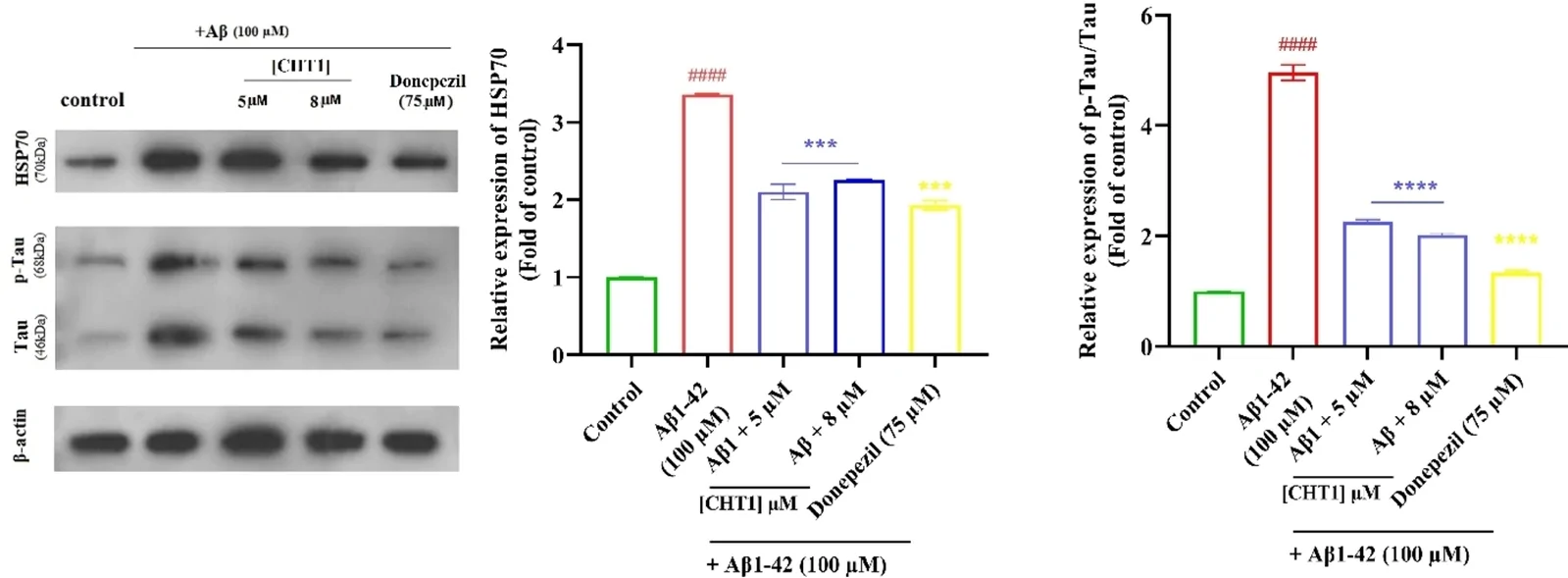
A Representative Western blot and quantification analysis of p‐Tau, Tau and HSP70 in PC12 cells pretreated with CHT1. Western blot analysis of HSP70, p‐Tau (68 kDa), Tau (46 kDa), and β‐actin in PC12 cells pretreated with CHT1 (5 and 8 μM) and exposed to Aβ1‐42 (100 μM). Donepezil (75 μM) was utilized as a positive control. The values are presented as the means±SDs (n=3). *P* values <0.05 were considered to indicate statistical significance. #p <0.05, ##p <0.01, ###p <0.001 vs Control; *p <0.05, **p <0.01, ***p <0.001 vs Aβ1‐42 (100 μM) represent a significant difference according to one‐way ANOVA. The original Western blot images are shown in Figures S22–S24 in Supporting Information File.

Under pathological AD conditions, tau protein becomes hyperphosphorylated, and it has been hypothesized that this hyperphosphorylation disrupts the microtubule‐stabilizing role of tau. According to our findings, CHT1 (5 and 8 μM) markedly decreased p‐Tau/Tau (2.26‐ and 2.01‐fold) and HSP70 (2.1‐ and 2.26‐fold) expression compared with that in the Aβ group. The p‐Tau/Tau (4.96‐fold) ratio and HSP70 (3.35‐fold) level were significantly greater in the Aβ group than in the control group. Additionally, the results obtained with donepezil (a positive control) showed that the p‐Tau/Tau and HSP70 expression levels decreased by 1.33‐fold and 1.92‐fold, respectively (Figure 4) compared with the Aβ group. The results showed that decreased HSP70 protein expression was related to a reduced p‐Tau/ Tau ratio, suggesting an improvement in AD conditions in PC12 cells induced by Aβ.

The original Western blot images are shown in Figures S22–S24 in Supplementary material.

Plaques, tau hyperphosphorylation, and neuronal cell death are the hallmark pathologic characteristics of AD, a common neurodegenerative disease. HSP70 is crucial for tau balance in neurons and is implicated in the pathogenesis of AD by regulating the phosphorylation, aggregation, and degradation of p‐Tau.^[38]^ HSP70 inhibits tau aggregation and enhances turnover, preventing toxic aggregates and tau‐related neurodegeneration.^[12]^ CHT1 was investigated in this study to determine whether it protects against Aβ1‐42 toxicity in PC12 neuronal cells. The findings revealed that CHT1, at a therapeutically relevant concentration, was able to protect and rescue neuronal cells from the harmful effects of the Aβ1‐42 peptide. The expression of p‐Tau, Tau, and HSP70 was analyzed via Western blotting to determine the impact of CHT1, and it was observed that exposure to CHT1 resulted in a decrease in the phosphorylation of tau and HSP70 expression caused by Aβ1‐42.

**Figure 5:**
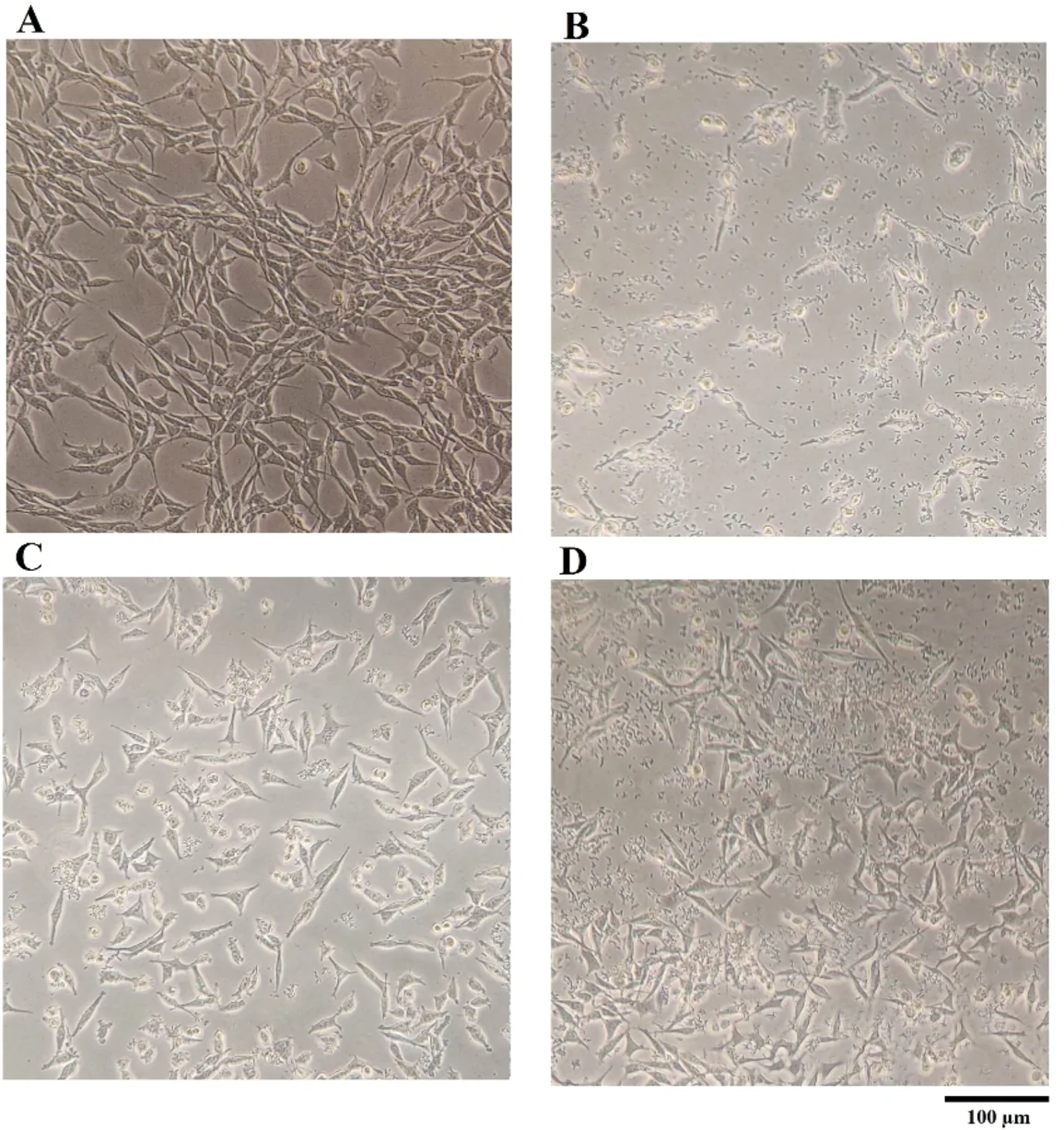
Growth of PC‐12 cells in response to various treatments. The growth of PC12 cells in (A) the control group and after exposure to (B) 100 μM Aβ, (C) 5 μM CHT1, or (D) 8 μM CHT1 for 24 hours.

Figure 5 shows the growth of PC12 cells over 24 hours in response to different treatments (control, 100 μM Aβ, 5 μM CHT1 and 8 μM CHT1). Compared with those in the control group, the number and density of PC12 cells in the Aβ (100 μM, Figure 5B) group were reduced (Figure 5A). Compared with Aβ (100 μM), CHT1 pretreatment (in the presence of Aβ) increased the density of PC12 cells at concentrations of 5 (Figure 5C) and 8 μM (Figure 5D).

### 2.3 In silico Studies

#### 2.3.1 BBB Permeability, ADME, and Toxicity

The ability of the TZD derivatives to pass through the blood–brain barrier (BBB) was predicted by employing the CBLigand‐BBB server (available at http://www.cbligand.org), and the score acquired based on the structure of CHT1 was 0.067. Compounds with a score higher than 0.02 can cross the BBB. The predicted results for the compounds are shown in Figures S16–S21 in Supplementary material. The descriptors that were relevant to drug‐likeness properties were calculated using the MProTox‐II online property prediction of small molecules toolkit (Table 1).

**Table 1:** Drug‐likeness properties of CHT1‐5.

Compounds	MW^[a]^	RB^[b]^	HA^[c]^	HD^[d]^	tPSA^[e]^	LogP (O/w)^[f]^
**CHT1**	235.26	2	4	1	80.7	2.35
**CHT2**	239.68	1	3	1	71.47	2.99
**CHT3**	221.23	1	4	2	91.7	2.04
**CHT4**	221.23	1	4	2	91.7	2.04
**CHT5**	225.29	1	3	1	79.75	3.49

[a] MW, Molecular Weight. [b] RB, number of Rotatable Bonds. [c] H.A, number of hydrogen acceptors. [d] HD, number of hydrogen donors. [e] tPSA, topological polar surface area. [f] logP, lipophilicity(Octanol/water).

In drug discovery development, the absorption, distribution, metabolism, and excretion of molecules (ADME) and toxicity may have a considerable impact on the success of a drug in clinical trials. Considering these factors is crucial when developing new treatments. To predict ADMET, an online property estimation toolkit called admetSAR estimates the pharmacokinetic parameters of synthesized TZD derivatives. The toolkit can be accessed online at http://lmmd.ecust.edu.cn/admetsar2. The predicted results of ADMET are shown in Table 2. The synthesized compounds in Table 2 have promising molecular characteristics that make them potential drug candidates.

**Table 2:** ADME and toxicity properties estimated for CHT1‐5.

Model	**CHT1**	**CHT2**	**CHT3**	**CHT4**	**CHT5**
Absorption					
Blood‐Brain Barrier	Positive	Positive	Positive	Positive	Positive
Human Intestinal Absorption	Positive	Positive	Positive	Positive	Positive
Caco‐2 Permeability	Positive	Negative	Negative	Negative	Negative
P‐glycoprotein Substrate	Nonsubstrate	Nonsubstrate	Nonsubstrate	Nonsubstrate	Nonsubstrate
Renal Organic Cation Transporter	Noninhibitor	Noninhibitor	Noninhibitor	Noninhibitor	Noninhibitor
Distribution					
Subcellular localization	Mitochondria	Mitochondria	Mitochondria	Mitochondria	Mitochondria
Metabolism					
CYP450 2D6 Inhibitor	Nonsubstrate	Nonsubstrate	Nonsubstrate	Nonsubstrate	Nonsubstrate
Toxicity					
AMES Toxicity	Non AMES toxic	Non AMES toxic	Non AMES toxic	Non AMES toxic	Non AMES toxic
Carcinogens	Non‐carcinogens	Non‐carcinogens	Non‐carcinogens	Non‐carcinogens	Non‐carcinogens
Rat Acute Toxicity	2.3412 (LD_50_, mol/kg)	2.5423 (LD_50_, mol/kg)	2.2784 (LD_50_, mol/kg)	2.2784 (LD_50_, mol/kg)	2.2198 (LD_50_, mol/kg)
Human Ether‐a‐go‐go‐Related Gene	Weak inhibitor	Weak inhibitor	Weak inhibitor	Weak inhibitor	Weak inhibitor

#### 2.3.2 Molecular Docking of CHT1 and Donepezil

The AChE enzyme has various subsites that have different functions. The AChE active site has two subsites – an anionic site and an esteratic site – which includes catalytic residues and is also referred to as the triad catalytic site.^[39]^ Apart from the active site, AChE also has a peripheral anionic site (PAS), an acyl‐binding site, and a choline‐binding site. AchE‐Is have an increasing affinity for these subsites, which play a crucial role in AD.^[40]^ Previous docking studies and Dvir et al. confirmed that tyrosine residues in the active‐site gorge are close to the catalytic site.^15^, ^26^, ^41^ Many studies have shown that aromatic residues play a vital role in AChE activity.^[42]^

**Figure 6:**
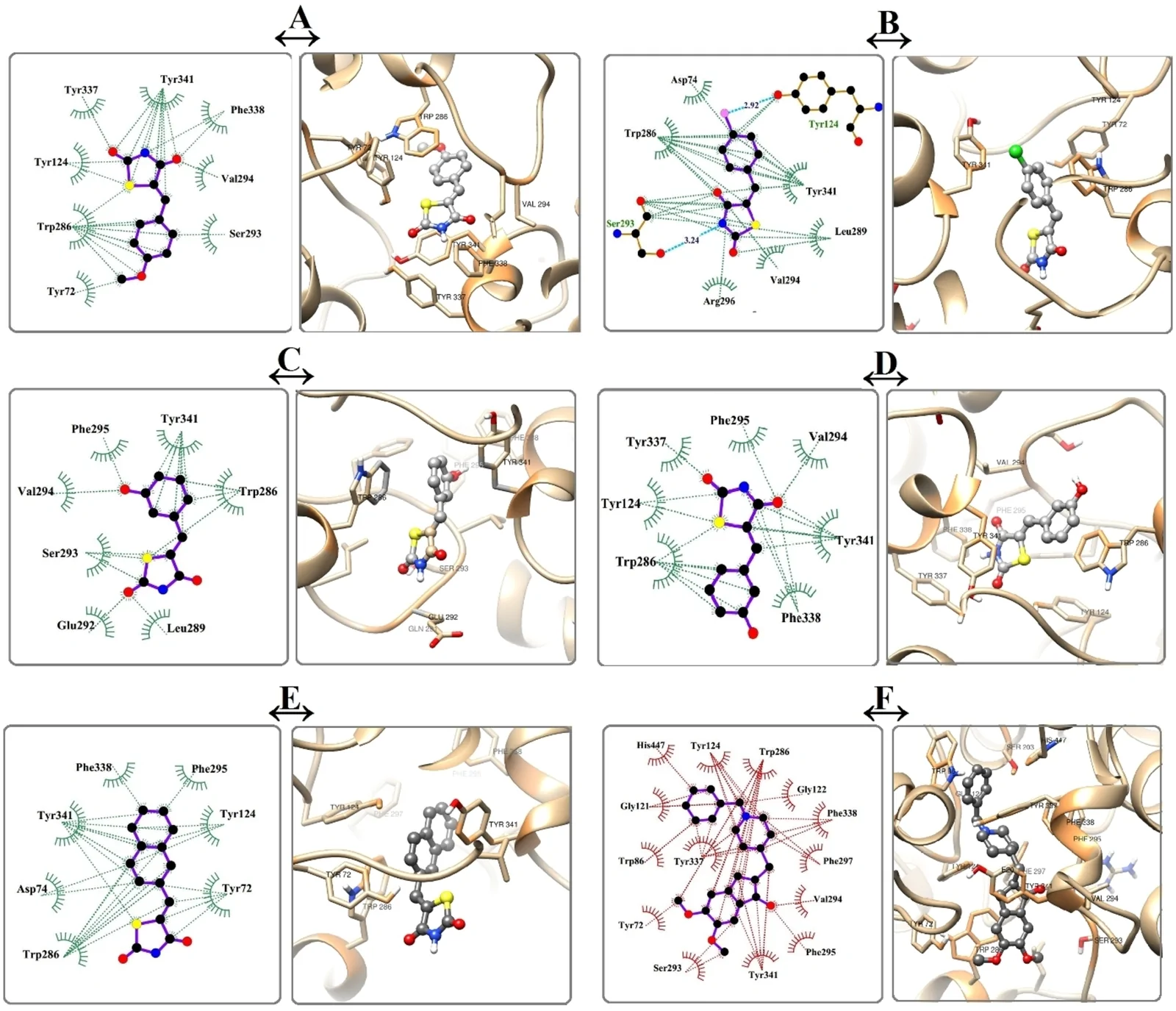
2D (left) and 3D (right) pictures of the molecular docking of TZD derivatives inside the gorge pocket of AChE. The most stable interactions between (A) CHT1, (B) CHT2, (C) CHT3, (D) CHT4, (E) CHT5, and (F) donepezil and AChE were determined by the number of residues involved in binding. The protein and ligands are depicted as solids and ribbons.

At the end of the docking process, the best conformation was determined, and the scoring functions revealed that it was the most appropriate complement to AChE. All docking calculations were performed using Autodock 4.2 software. To validate the docking results obtained using Autodock, donepezil in the active site of AChE was docked as a positive control ligand with an affinity of −7.43 kcal/mol. The most commen residues associated with the TZD derivative and donepezil were Tyr 124, Tyr 72, Tyr341, Tyr 337, Phe 295, Phe 338, Trp 286, Ser 293, and Val 294 in the AChE active site. The binding free energy of the docking of CHT1 to the active site was computed to be −8.25 kcal/mol. CHT1 and donepezil interacted with the Trp 286, Val 294, Tyr 72, and Ser 293 residues in AChE, which conform to the PAS of the active site of AChE. In addition, CHT1 formed a hydrogen bond with Tyr 341. The interactions between the formyl oxygen atom of the thiazol ring and Tyr 337 and Phe 338 and beetween the hydrogen and oxygen of the methoxy group of CHT1 and Trp 286 and Tyr 72 are illustrated in Figure 6. Sicak et al. indicated that Trp 286 and Tyr337 are responsible for the catalytic functional unit of AChE, which interacts with thiazolidin‐4‐one responsive derivatives.^[42b]^ In another study, Kua et al. confirmed that Tyr 337, and Trp 286 are important residues in the active site of AChE.^[43]^

Table 3 shows the binding free energy results and the amino acids implicated in hydrogen bonding and hydrophobic interactions with, CHT1‐5 and donepezil serving as positive controls for AChE. 3D and 2D images of the molecular docking of CHT1‐5 and donepezil (as a positive control) with AChE are shown in Figure 6. 3D and 2D interaction maps were prepared with UCSF Chimera^[44]^ and LigPlot,^[45]^ respectively.

**Table 3:** Binding free energy calculation results for TZD derivatives docked with AChE (PDB ID: 4 M0E) and a list of amino acids that interact via hydrogen bonding and hydrophobic interactions with TZD derivatives and donepezil (a positive control) with AChE.

Compounds	Residues involved in hydrophobic interactions	Hydrogen‐bond	Free Energy of Binding (kcal/mol)
**CHT1**	Tyr124, Tyr72, Tyr341, Tyr337, Val294, Trp286, Ser293	Tyr341	−8.25
**CHT2**	Tyr341, Tyr337, Trp286, Phe338, Leu289, Val294, Asp74, Ser293, Arg296	Ser293, Arg296, Tyr341	−7.26
**CHT3**	Trp286, Tyr341, Tyr124, Ser293, Val294, Phe295, Phe338, Arg296, Leu289	Tyr124, Ser293, Arg296, Phe295	−7.63
**CHT4**	Tyr341, Tyr124, Tyr334, Tyr337, Arg296, Leu289, Phe338, Phe295, Val294, Trp286	Phe295, Ser293	−7.46
**CHT5**	Trp286, Tyr72, Tyr341, Tyr124, Tyr337, Tyr241, Asp74, Phe295, Phe338, Val294,	Tyr341	−8.92
Donepezil	Tyr341, Tyr124, Tyr72, Ser293, Gly121, Gly122, Gly342, Gln291, Val294, Leu289, His447, Phe295, Phe297, Phe338, Val294, Trp86, Trp286	Trp286	−7.43

## 3 Conclusions

This study examined five newly synthesized compounds (CHT1–CHT5) and evaluated their effects. FT‐IR, ^1^H, and ^13^C NMR were used to analyze and identify the TZD derivatives. CHT1 was chosen for subsequent studies based on the results of FRAP assays. According to the investigation, CHT1 was efficacious in inhibiting AChE, with an IC_50_ of 165.93 nM. These findings imply that CHT1 has the potential to be a valuable medicinal agent for treating conditions associated with AChE inhibition. CHT1 protects PC12 neural cells from the toxicity of the Aβ1‐42 peptide. Additional investigations involving Western blot analysis have indicated that CHT1 may have promising therapeutic potential. Specifically, the compound has been shown to reduce the expression of p‐Tau/Tau, which is a significant contributing factor to the development of Alzheimer's disease. Furthermore, the level of HSP70 expression decreased, which may indicate a decrease in the pathological symptoms associated with the disease. To fully comprehend the mechanism of efficacy and potential medical applications of CHT1, further study is needed since it has been shown to reduce the expression of p‐Tau/Tau and HSP70.

The combined influence of the electron‐rich methoxy group and the TZD moiety in TZD derivatives significantly enhances the inhibition effect. Replacing the methoxy group with a hydroxy group in CHT3 and CHT4 enhanced activity, leading to CHT1 exhibiting significantly better inhibition against ChEs. According to these findings, a methoxy group at the para position of the phenyl ring could be a valuable therapeutic agent under treatment conditions related to AChE inhibition. Due to its multifunctional properties, CHT1 is a promising candidate for fresh studies on the impacts of novel drugs on Alzheimer's disease, which is an optimal strategy. Conducting thorough research before using a new therapeutic agent is essential.

## Conflict of Interests

The authors declare no conflict of interest.

## Supporting information

As a service to our authors and readers, this journal provides supporting information supplied by the authors. Such materials are peer reviewed and may be re‐organized for online delivery, but are not copy‐edited or typeset. Technical support issues arising from supporting information (other than missing files) should be addressed to the authors.

Supporting Information

## Data Availability

The data that support the findings of this study are available from the corresponding author upon reasonable request.
