# Supplementary material for: Investigation of Thiazolidine‐2,4‐Dione Derivatives as Acetylcholinesterase Inhibitors: Synthesis, In Vitro Biological Activities and In Silico Studies
Source: ChemistryOpen. 2025 Jan 10;14(5):e202400294. doi: 10.1002/open.202400294 (PMC13580833; doi:10.1002/open.202400294)
Supplement: Supplementary file 1 [file OPEN-14-e202400294-s001.pdf]

ChemistryOpen

Supporting Information

Investigation of Thiazolidine-2,4-Dione Derivatives as Acetylcholinesterase Inhibitors: Synthesis, *In Vitro* Biological Activities and *In Silico* Studies

Hanane Naeimi, Maryam Taheri, Hossein Ghafouri,* and Asadollah Mohammadi

Supporting information contents

Figures S1-S15: FT-IR, ^{1}H NMR, and ^{13}C NMR spectra of CHT1, CHT2, CHT3, CHT4 and CHT5.

Figures S16-S21: Diagram of the ability of CHT1, CHT2, CHT3, CHT4, CHT5, and donepezil to pass through the BBB.

Figures S22-S24: Original photos of Western blot gels of HSP70, p-Tau and Tau, and β-actin.

FT-IR, ^{1}H NMR, and ^{13}C NMR spectra of CHT1, CHT2, CHT3, CHT4 and CHT5

5-(4-methoxybenzylidene)thiazolidine-2,4-dione (CHPT1)

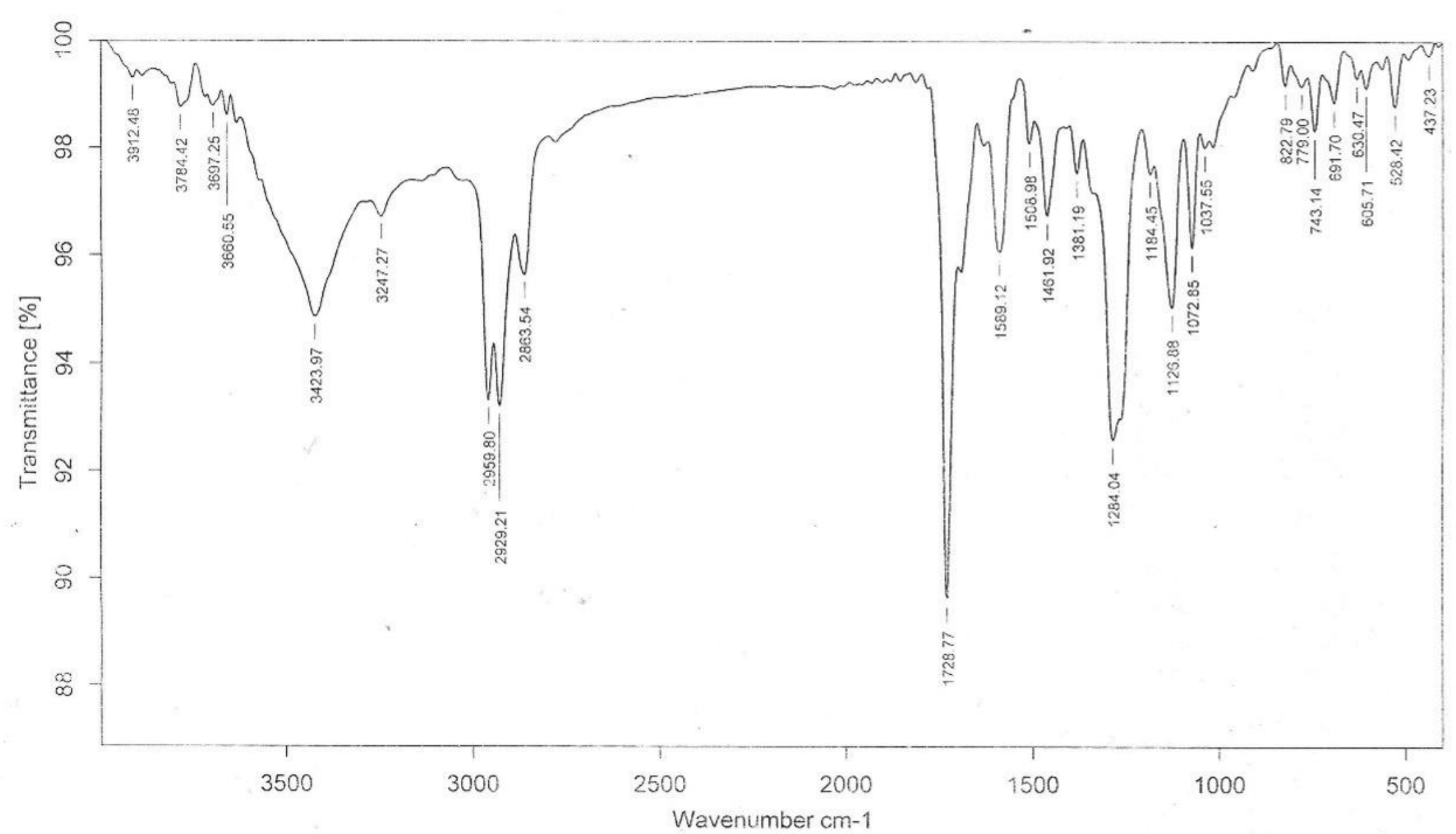

Figure S1. FT-IR spectra of CHT1.

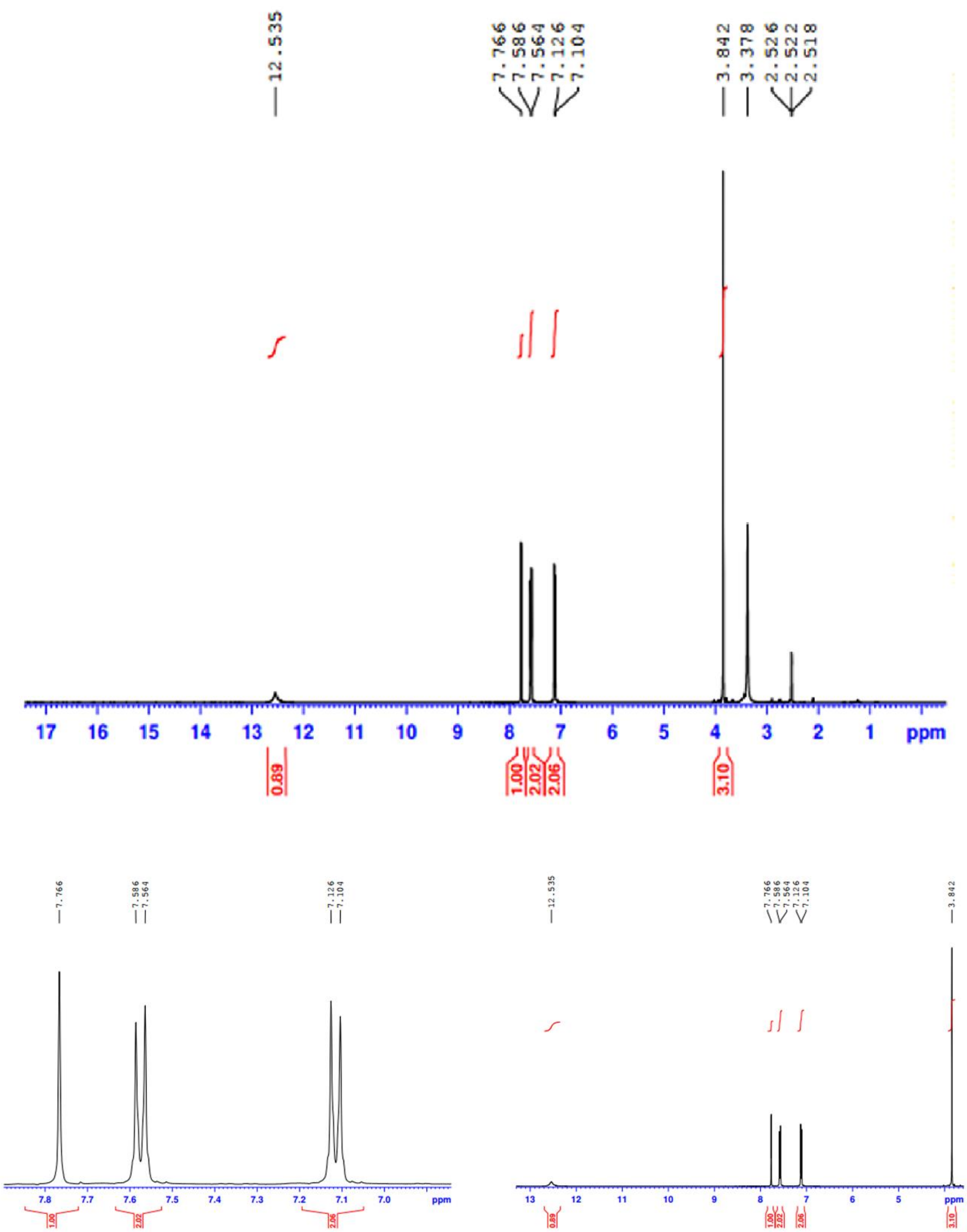

Figure S2. ^{1}H NMR spectra of CHT1.

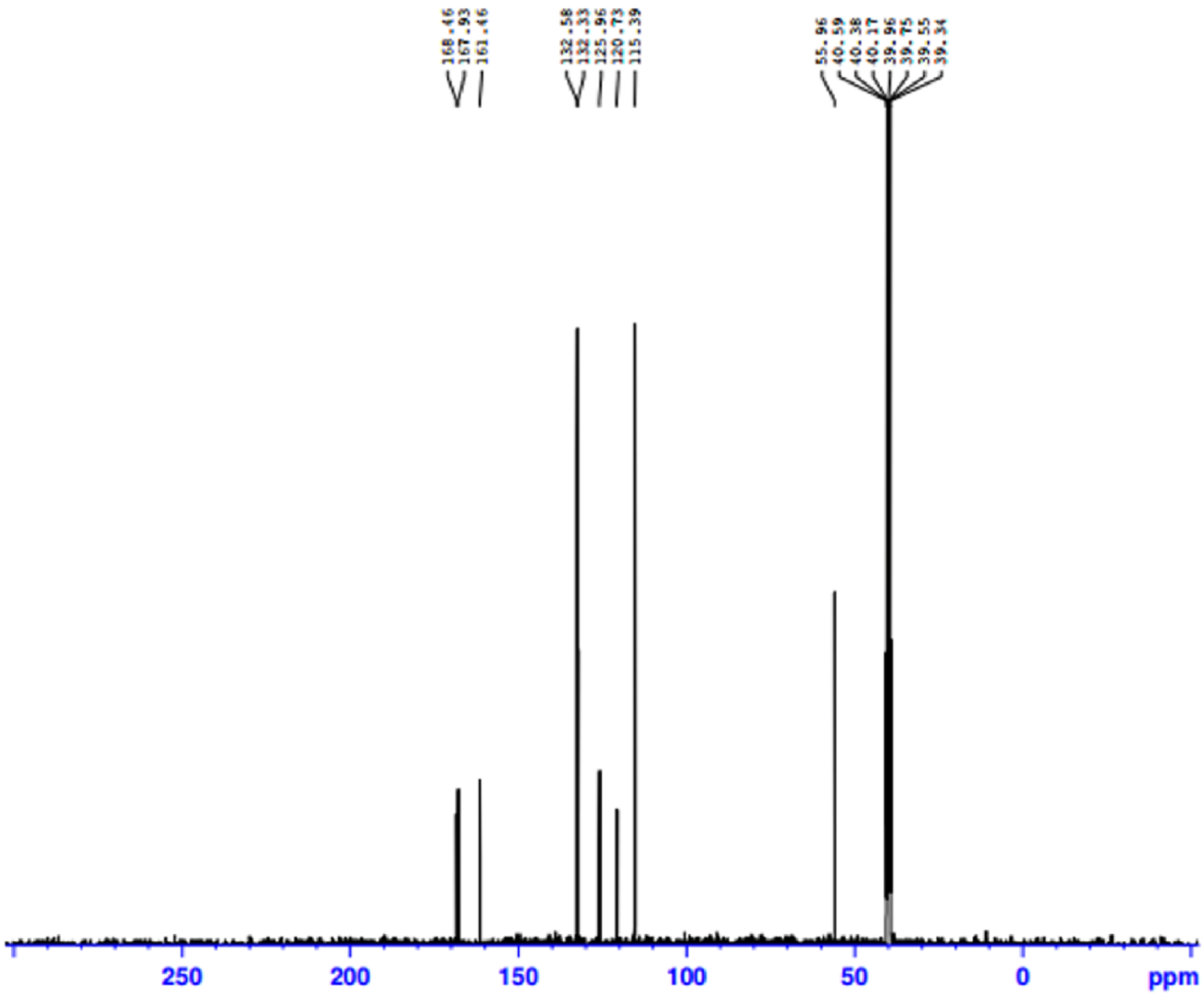

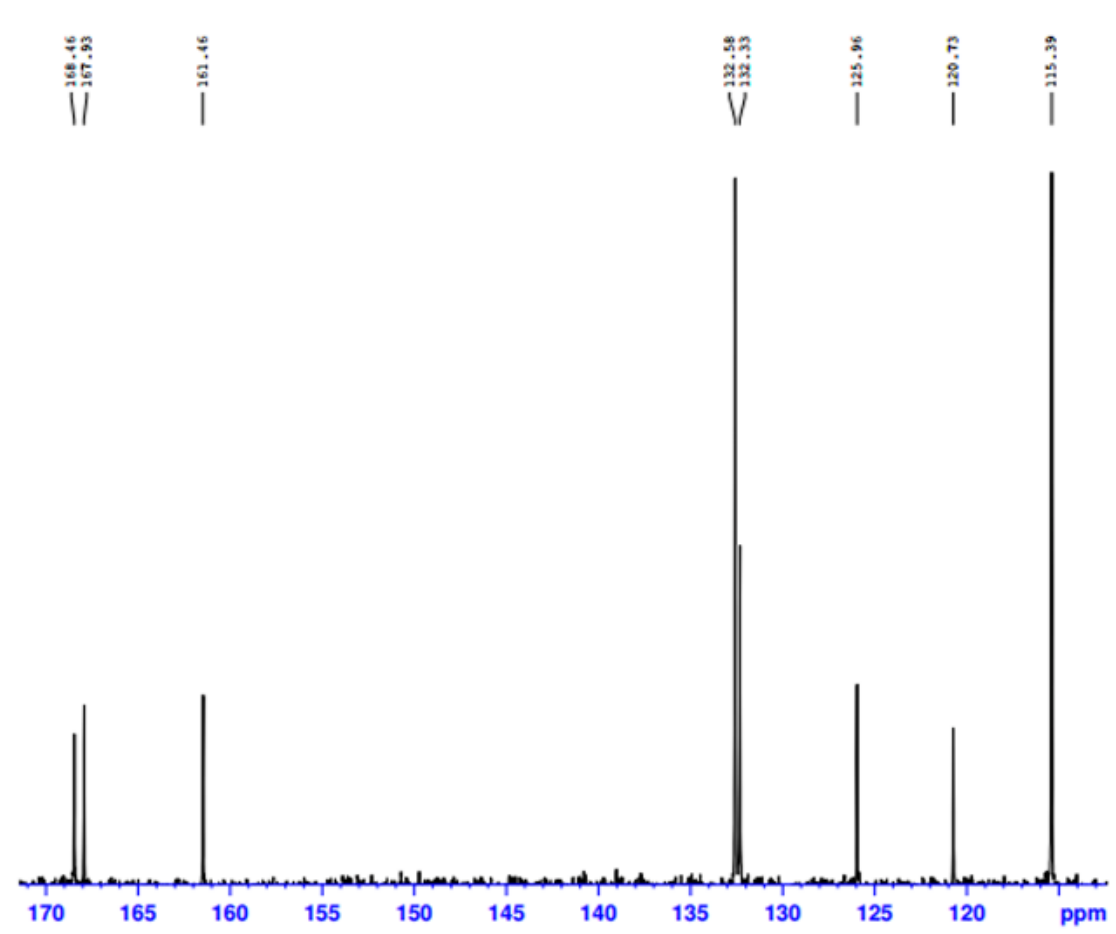

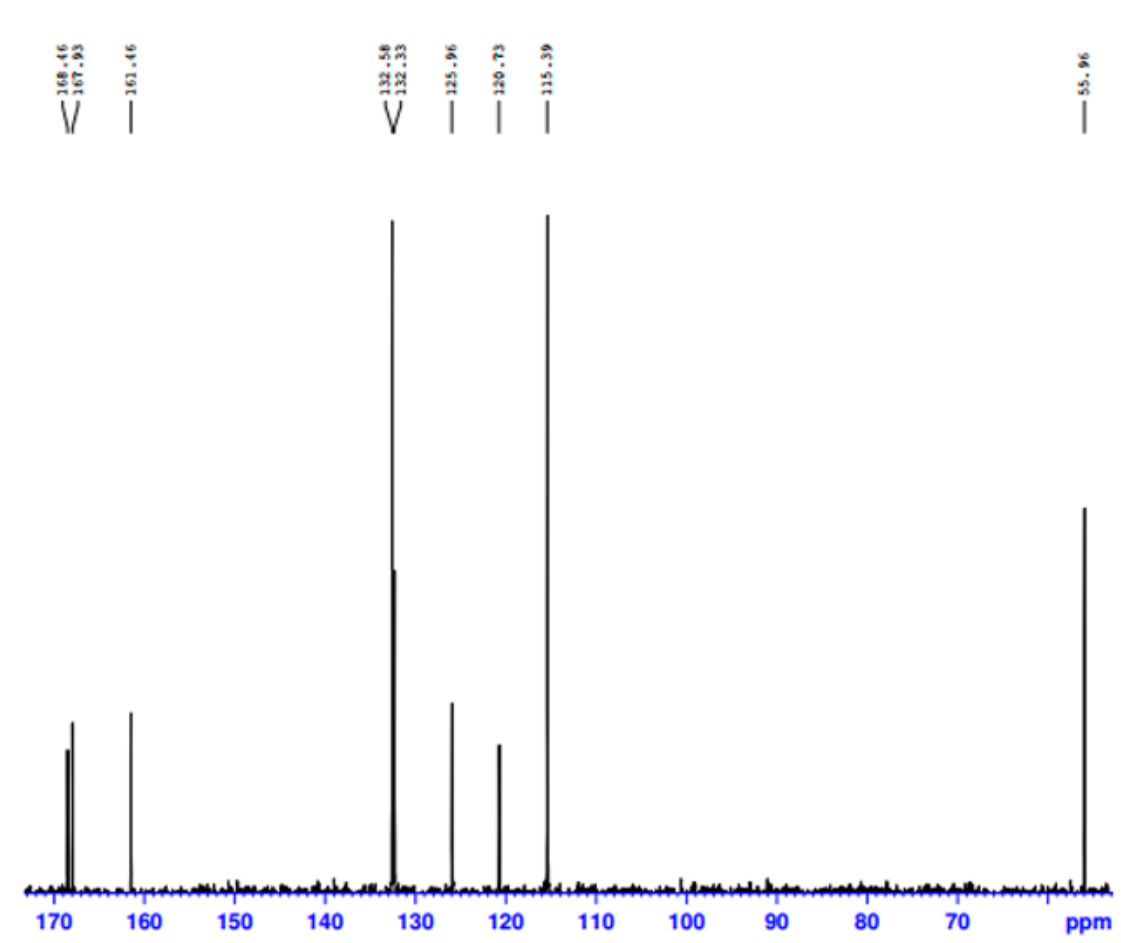

Figure S3. ^{13}C NMR spectra of CHT1.

5-(4-chlorobenzylidene) thiazolidine-2,4-dione (CHT2)

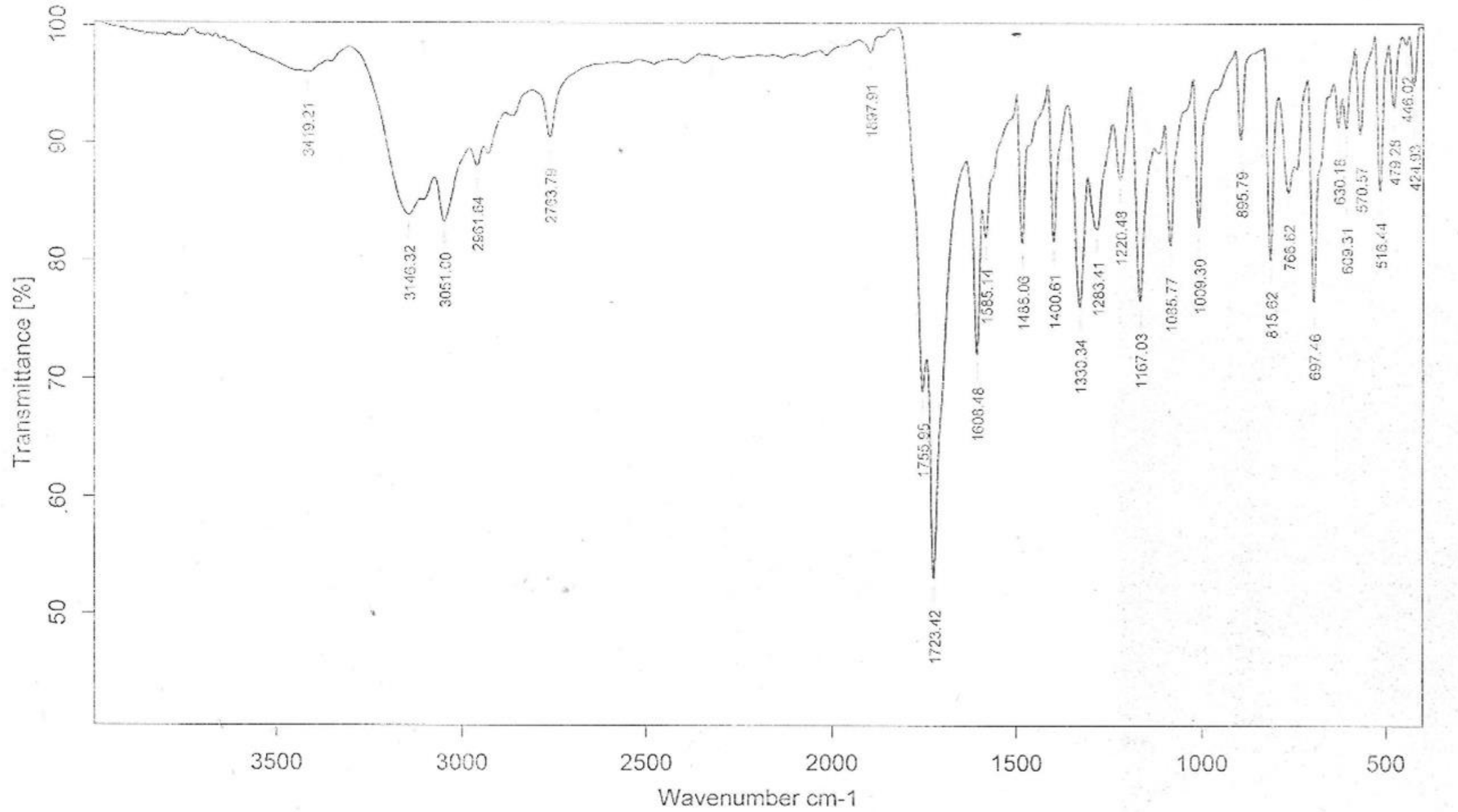

Figure S4. FT-IR spectra of CHT2.

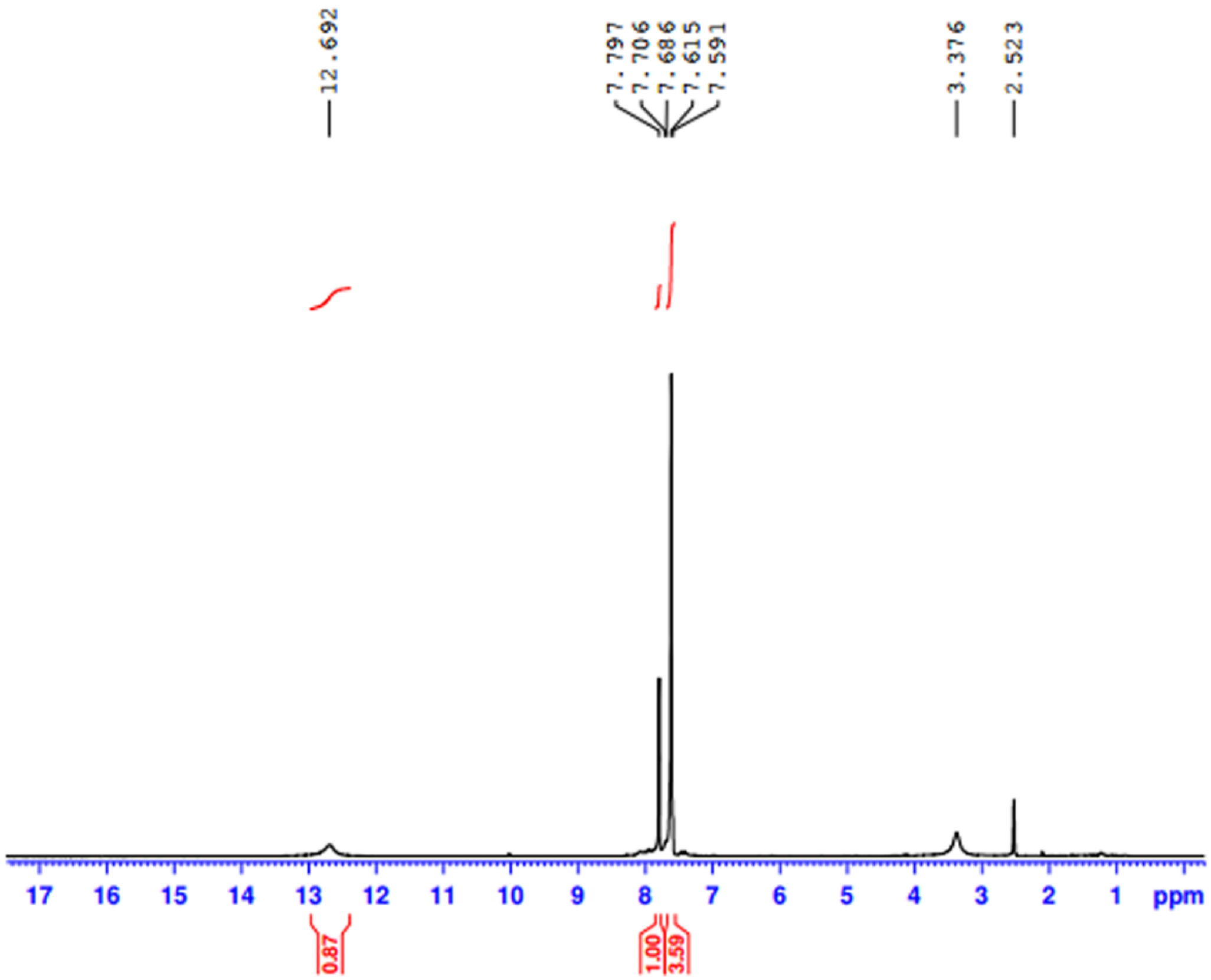

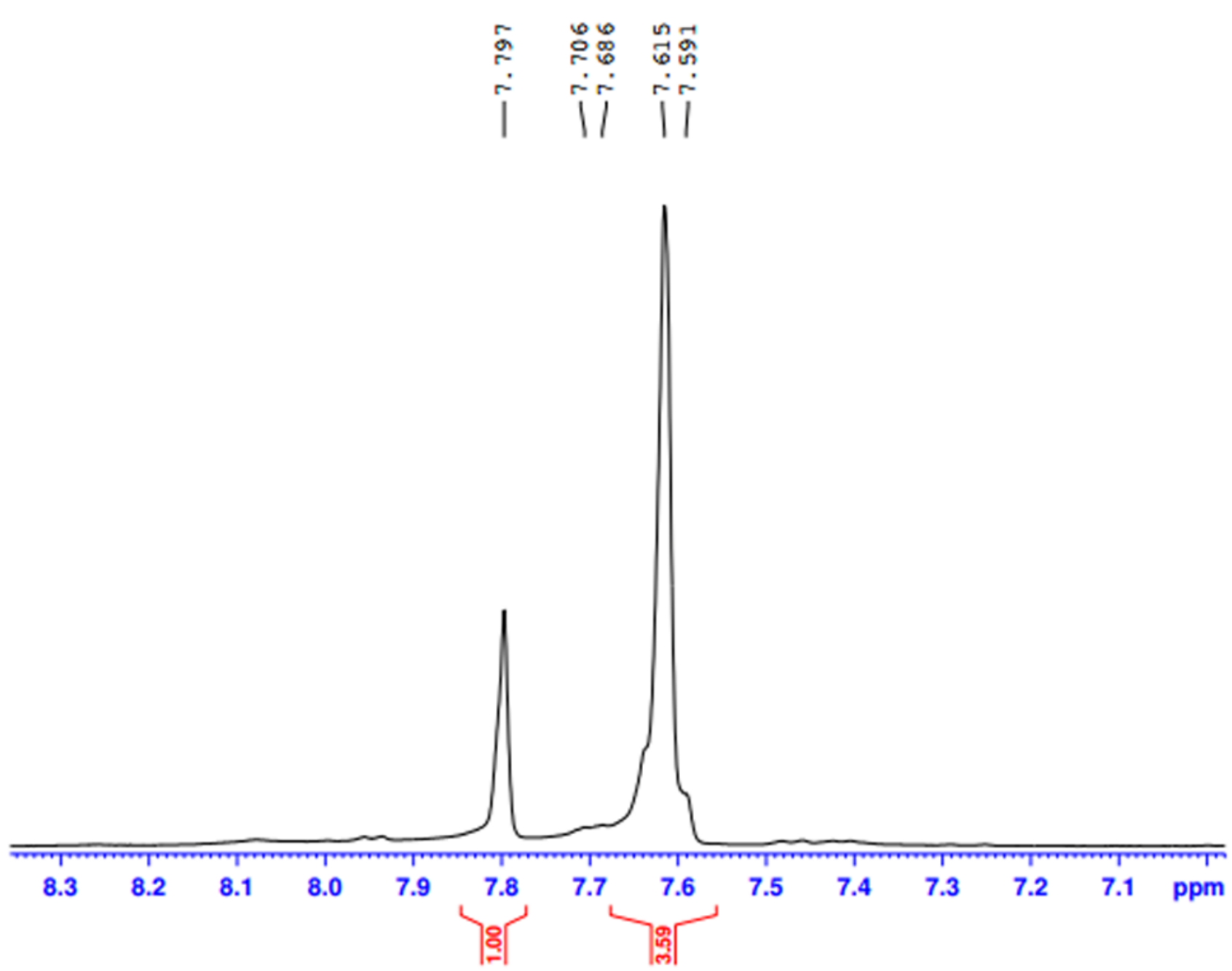

Figure S5. ^{1}H NMR spectra of CHT2.

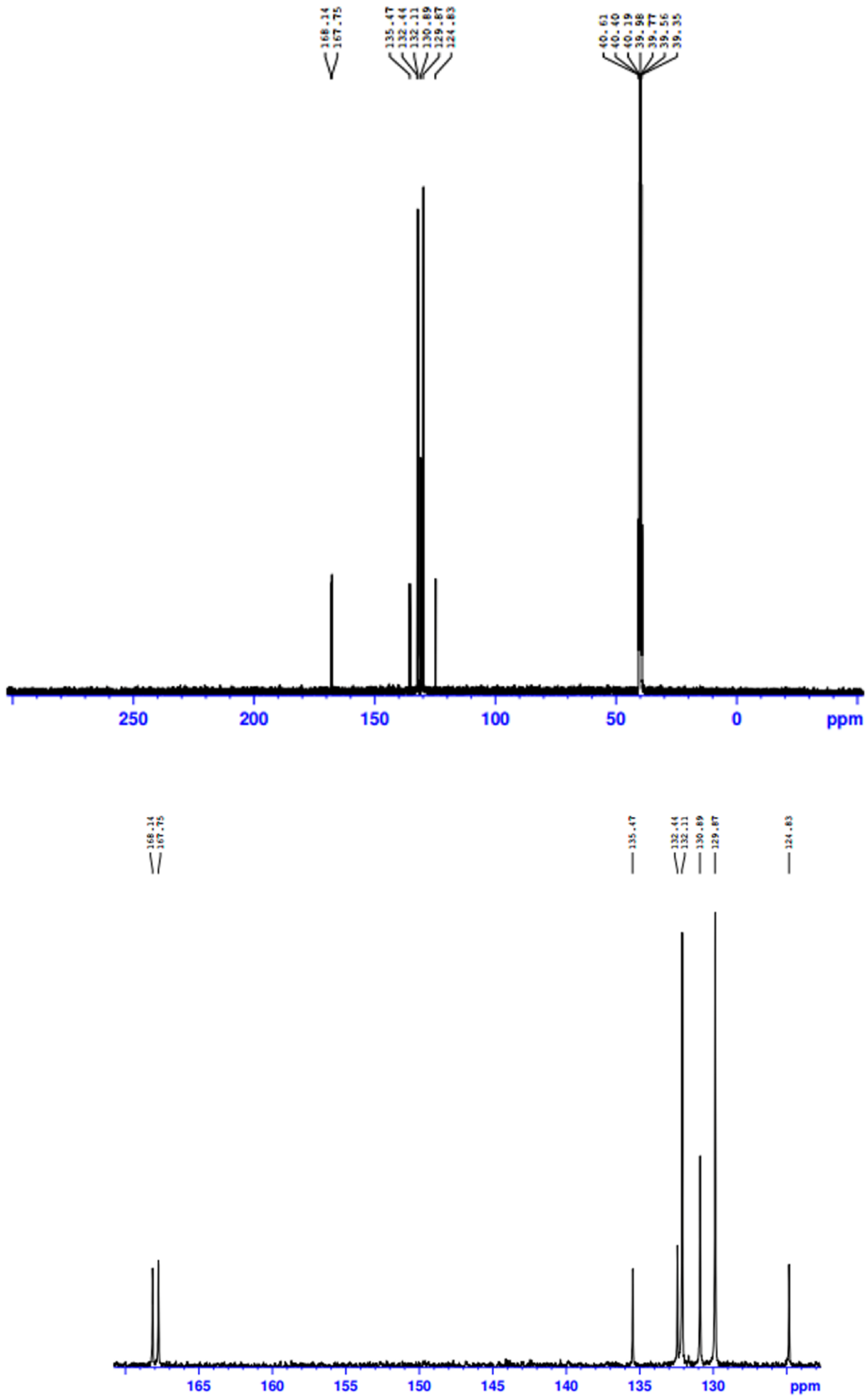

Figure S6. ^{13}C NMR spectra of CHT2.

5-(3-hydroxybenzylidene)thiazolidine-2,4-dione (CHT3)

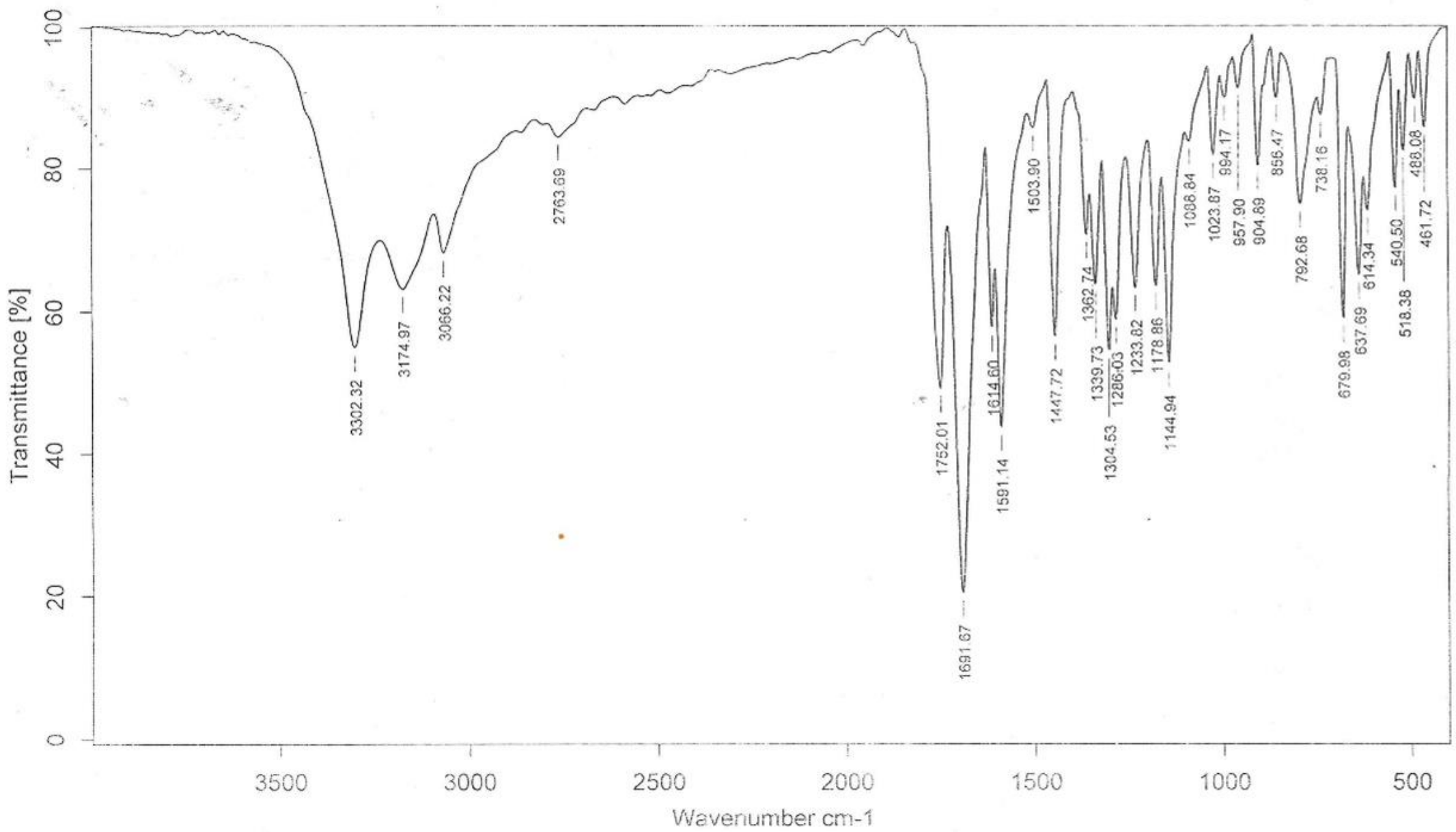

Figure S7. FT-IR spectra of CHT3.

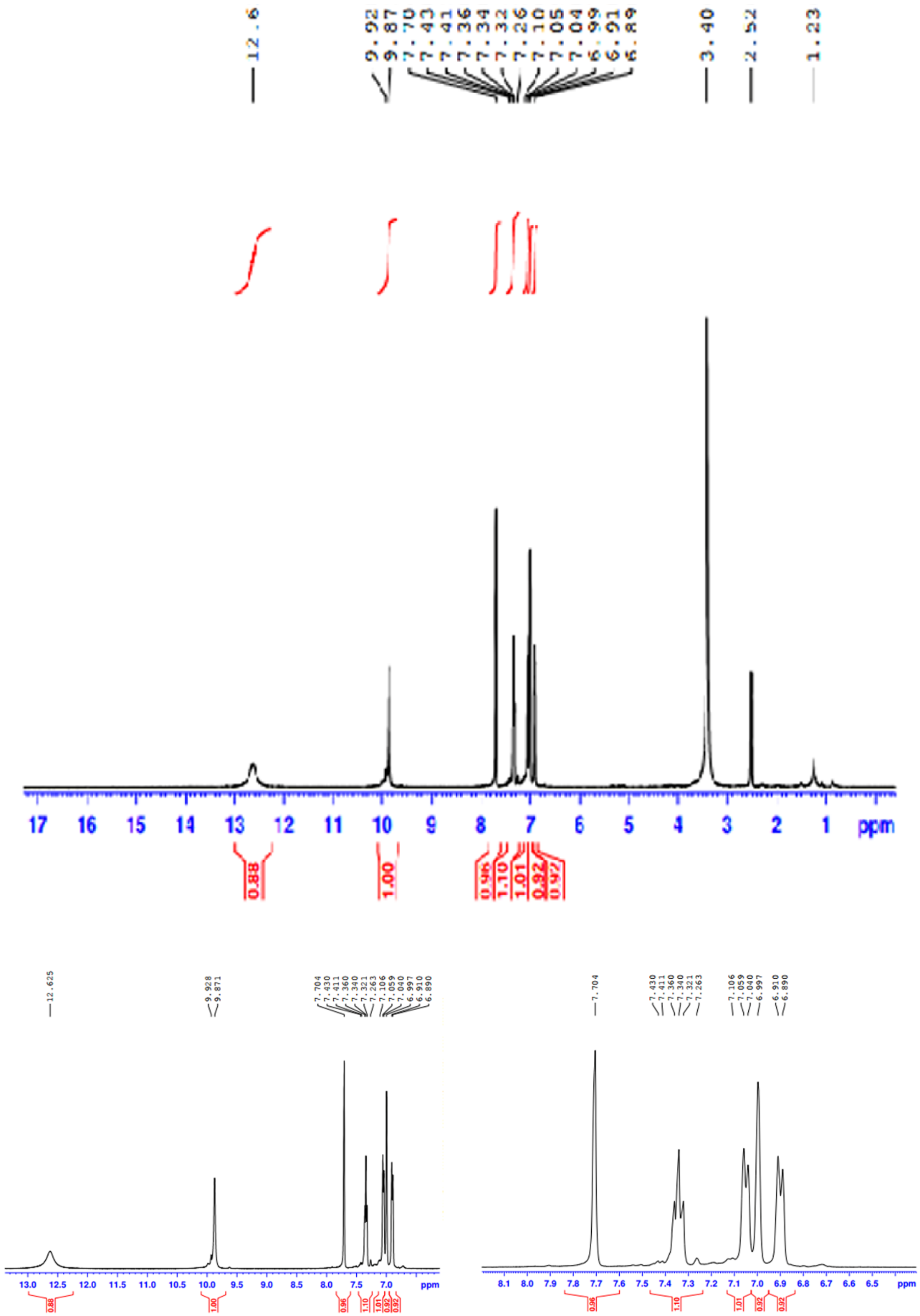

Figure S8. ^{1}H NMR spectra of CHT3.

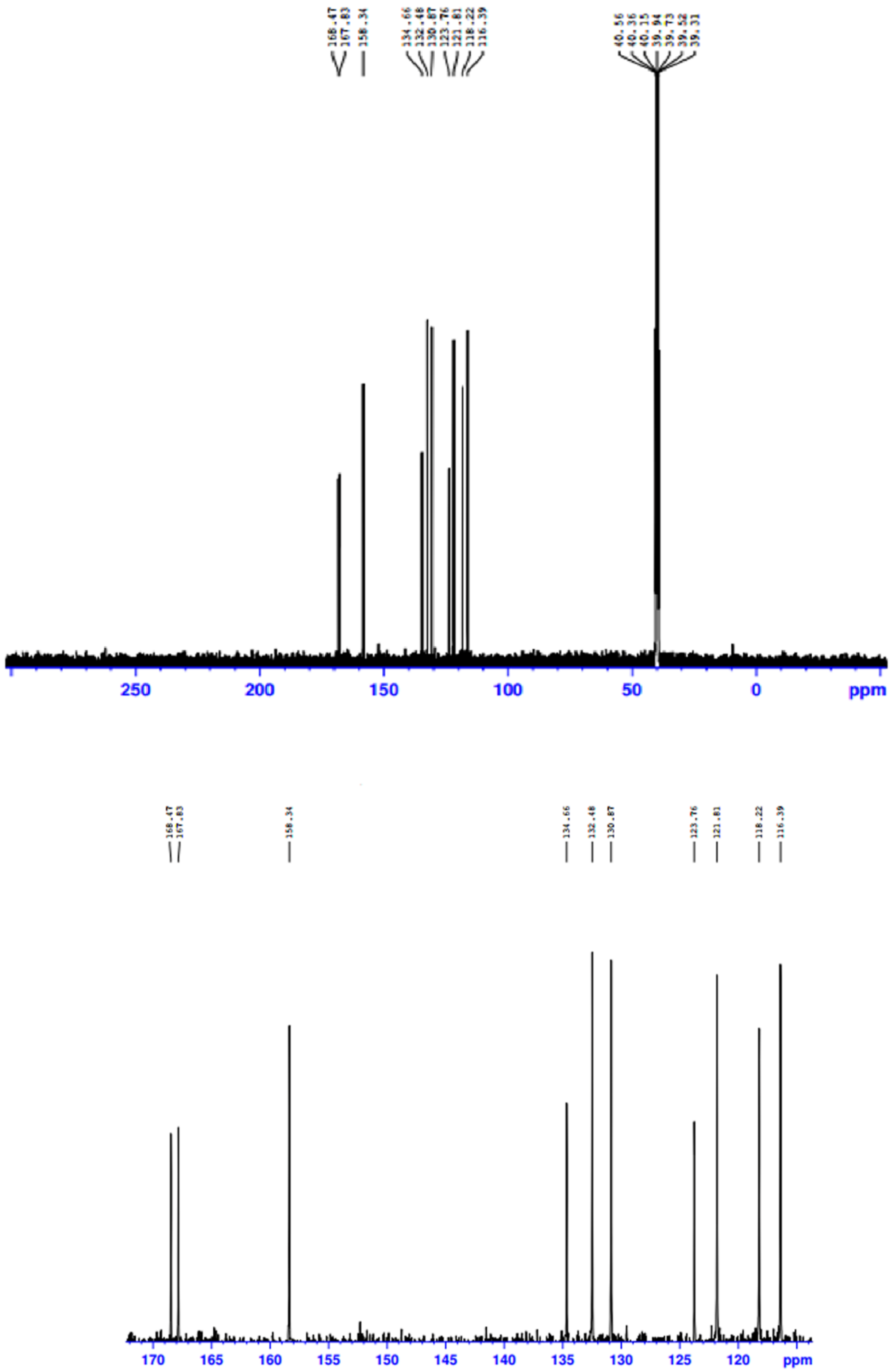

Figure S9. ^{13}C NMR spectra of CHT3.

5-(2-hydroxybenzylidene)thiazolidine-2,4-dione (CHT4)

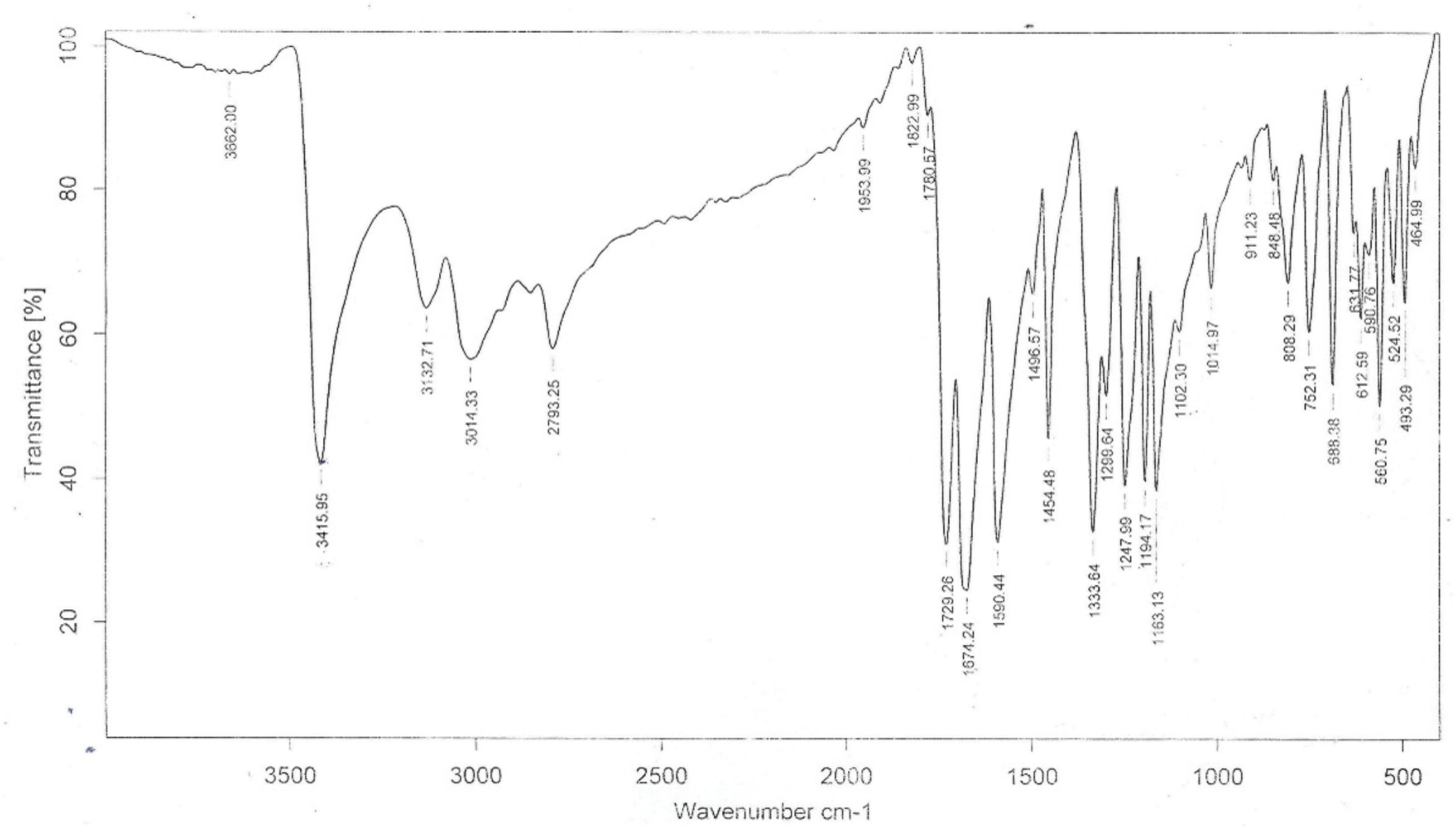

Figure S10. FT-IR spectra of CHT4.

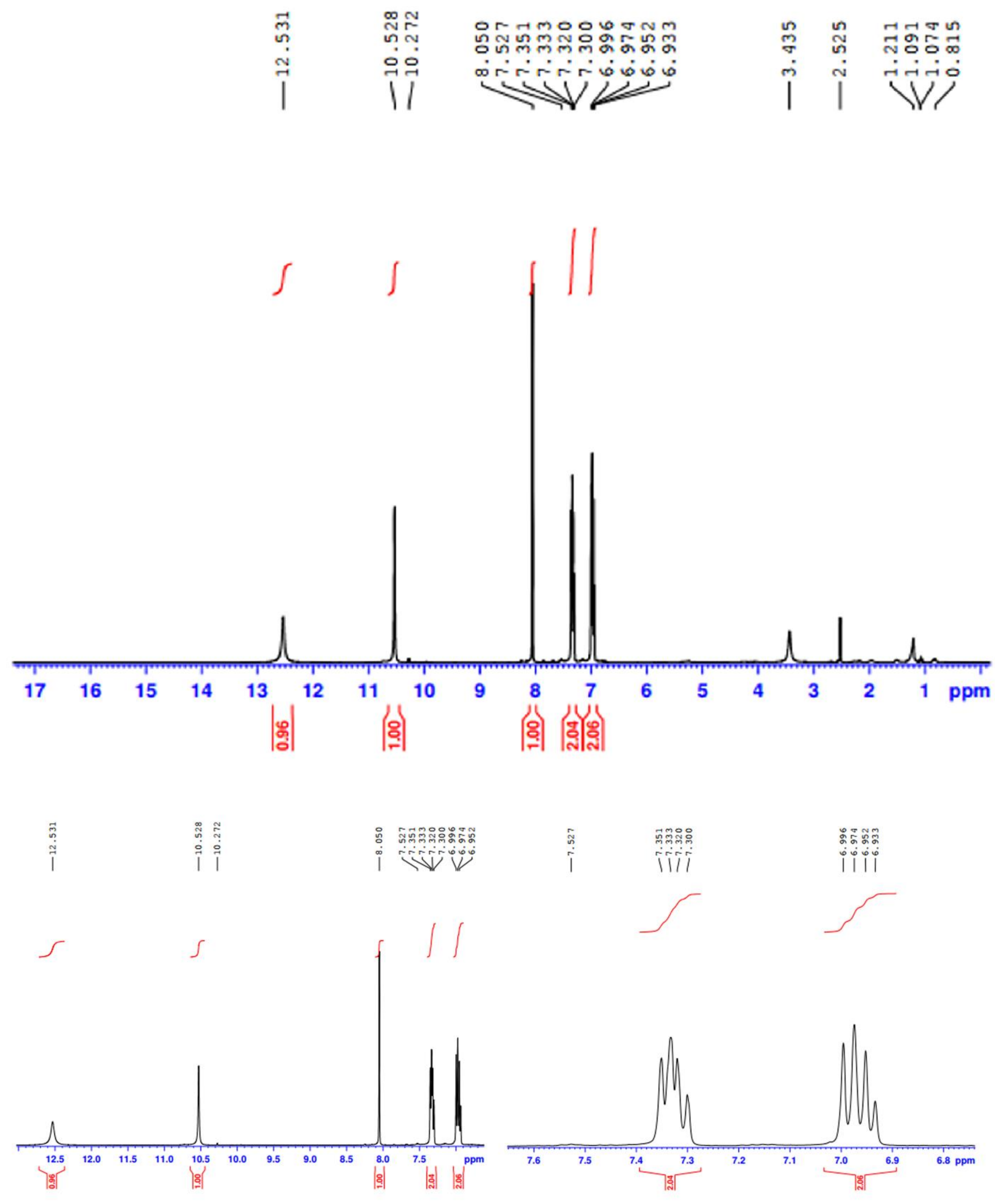

Figure S11. ^{1}H NMR spectra of CHT4.

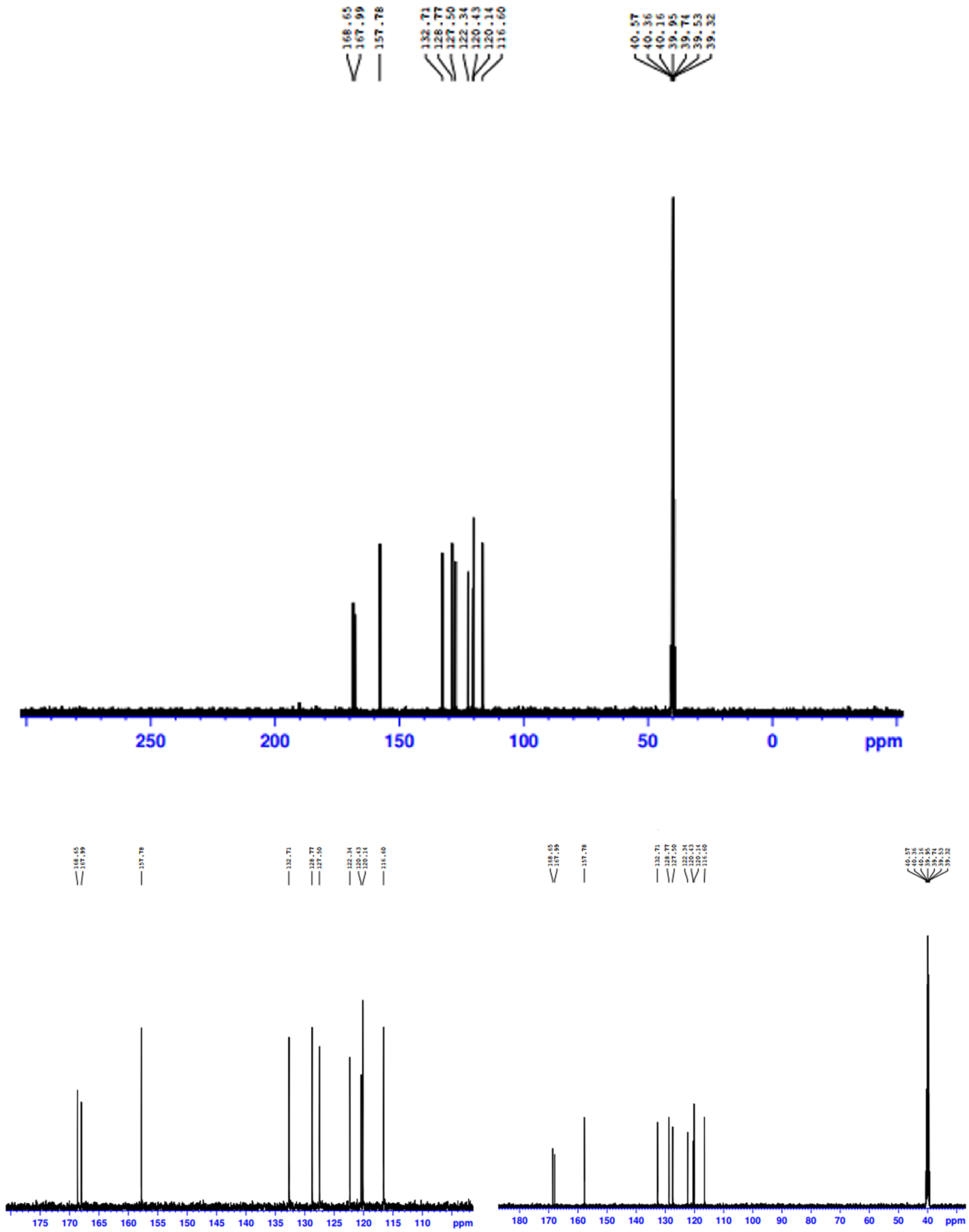

Figure S12. ^{13}**C NMR spectra of CHT4.**

5-((naphthalene-1-yl)methylene)thiazolidine-2,4-dione (CHT5)

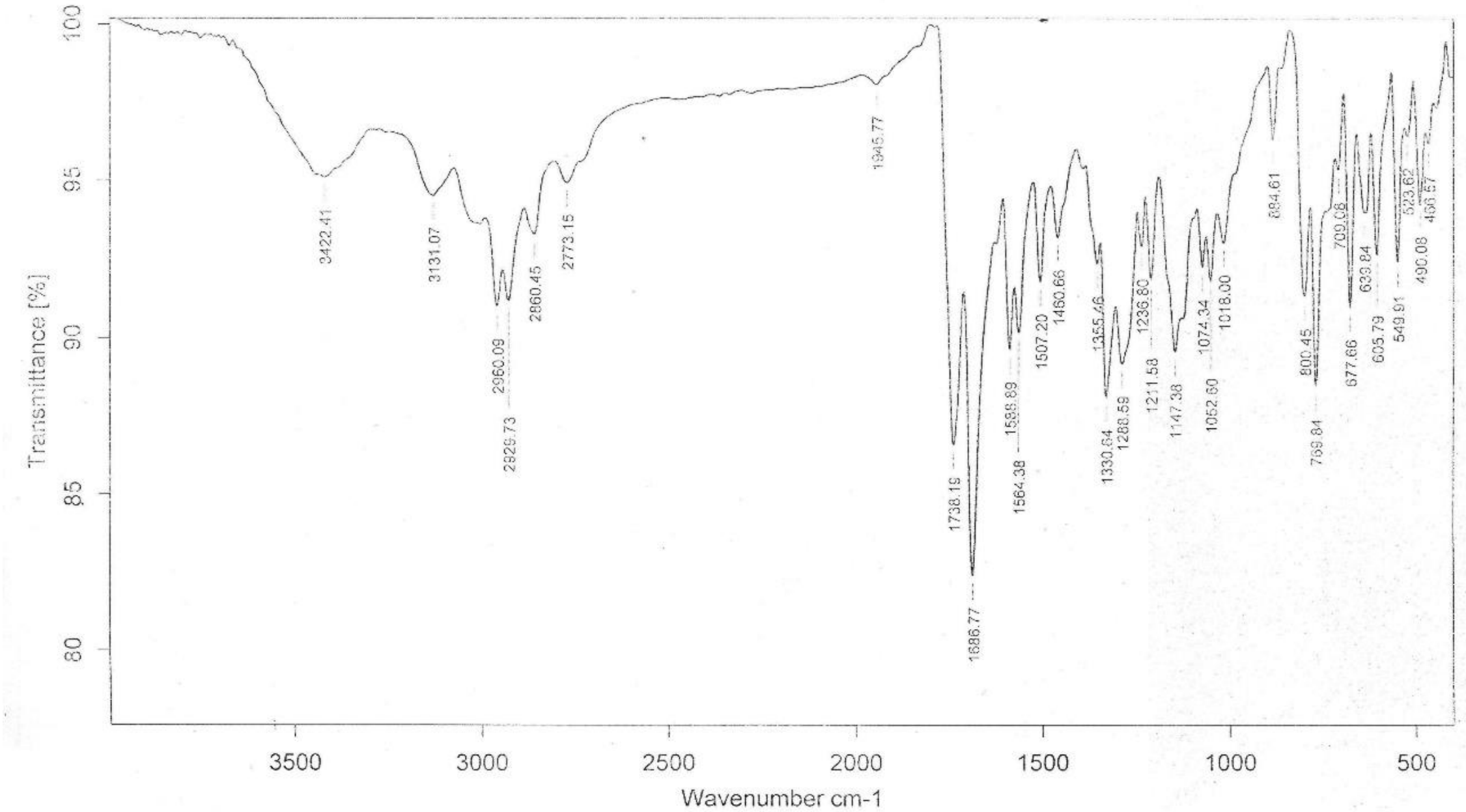

Figure S13. FT-IR spectra of CHT5.

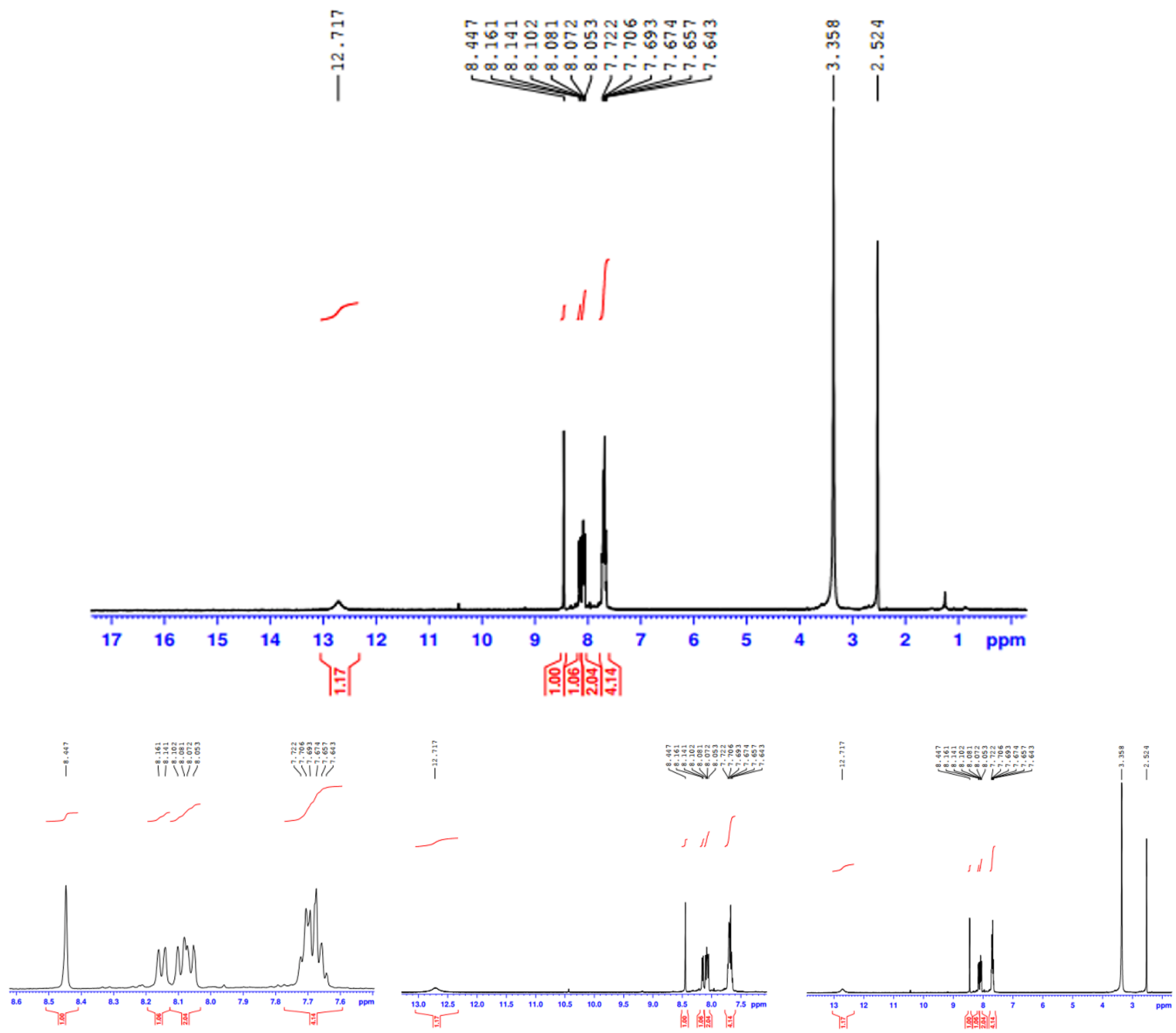

Figure S14. ^{1}H NMR spectra of CHT5.

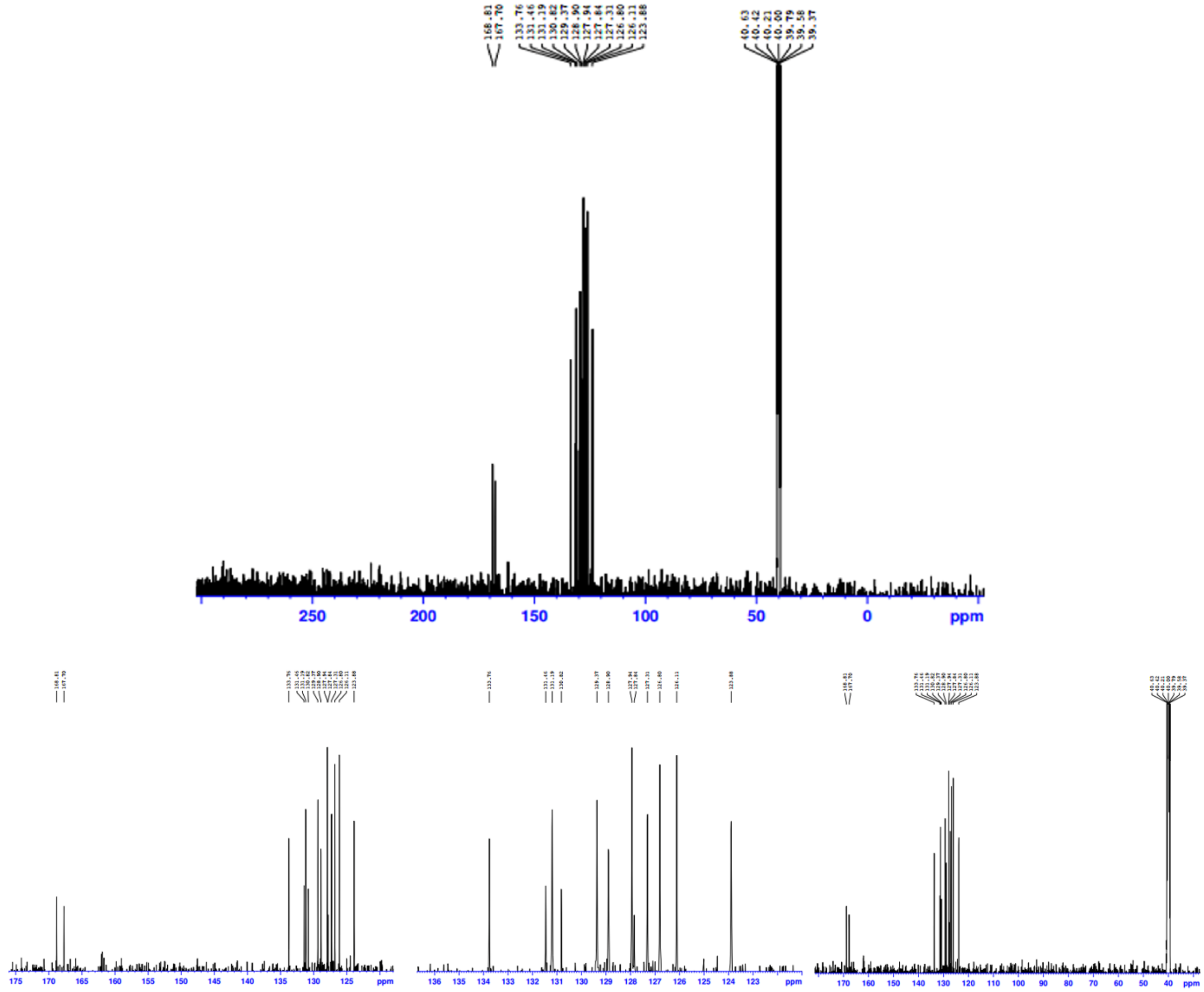

Figure S15. ^{13}C NMR spectra of CHT5.

Diagram of the capability of CHT CHT1, CHT2, CHT3, CHT4, CHT5, and donepezil in passing from BBB

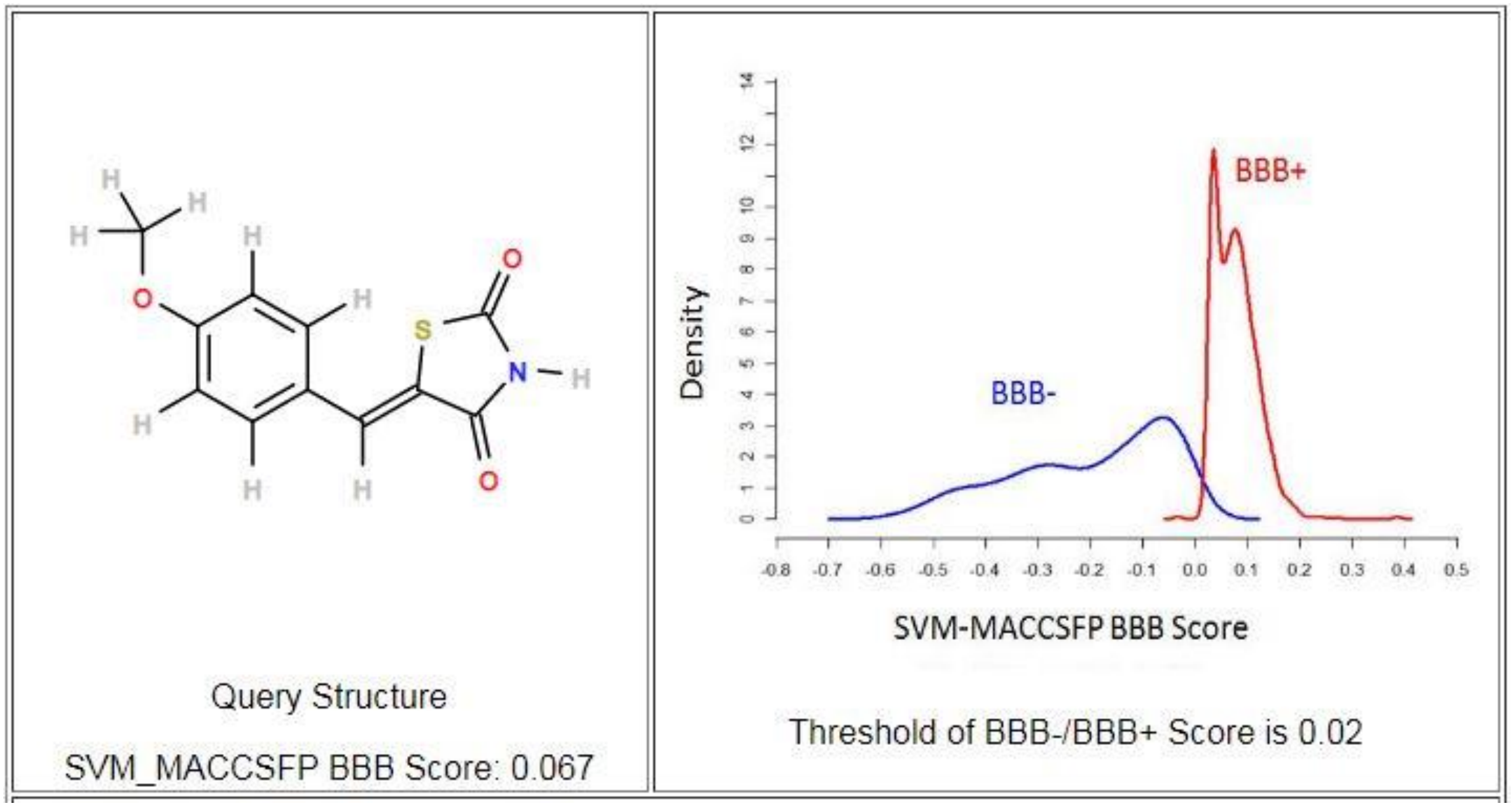

Figure S16. Diagram of the capability of the CHT1 in passing from BBB. The passage of compound CHT1 was predicted through the blood-brain barrier using the BBB Predictor server.

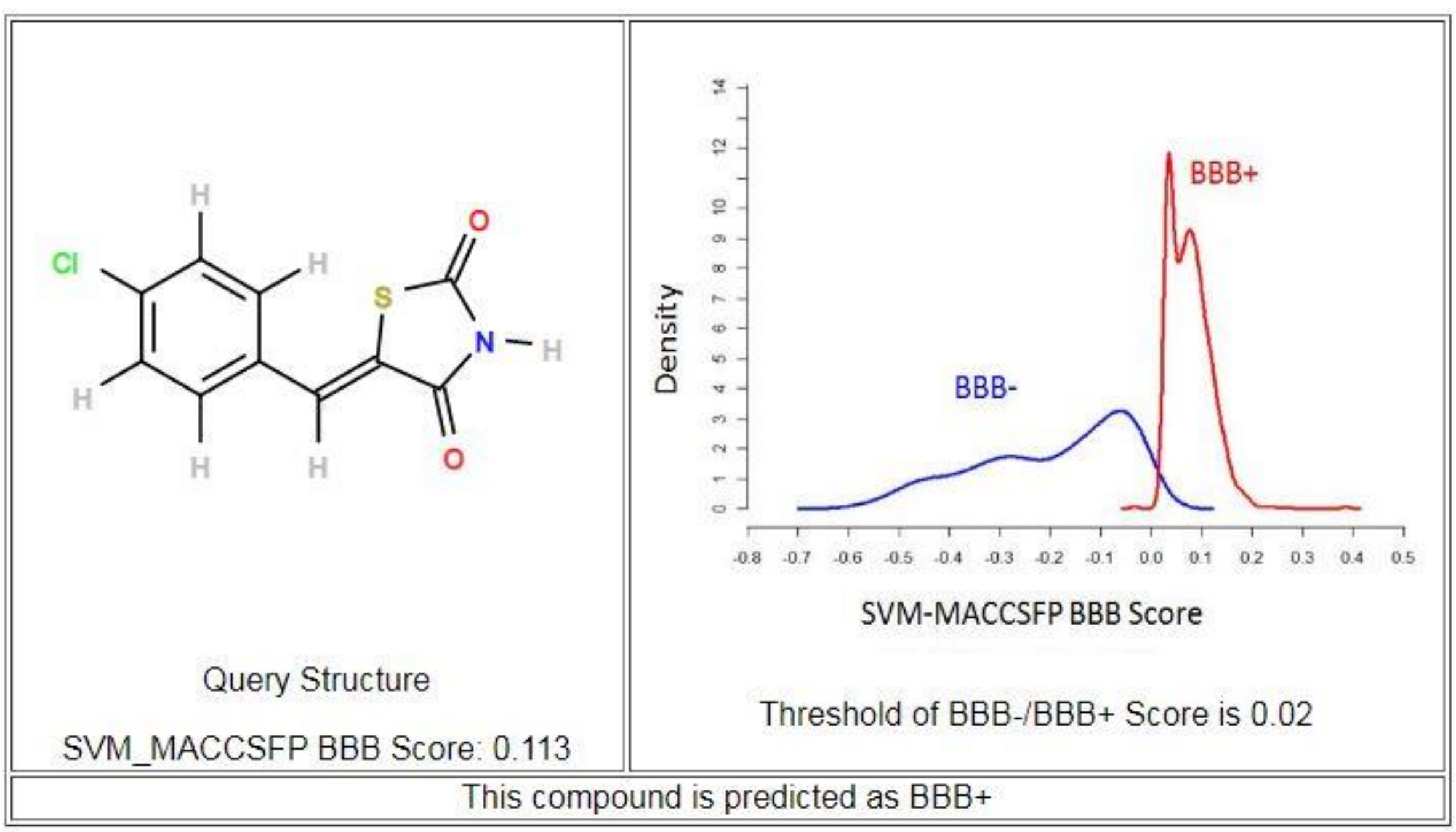

Figure S17. Diagram of the capability of the CHT2 in passing from BBB. The passage of compound CHT2 was predicted through the blood-brain barrier using the BBB Predictor server.

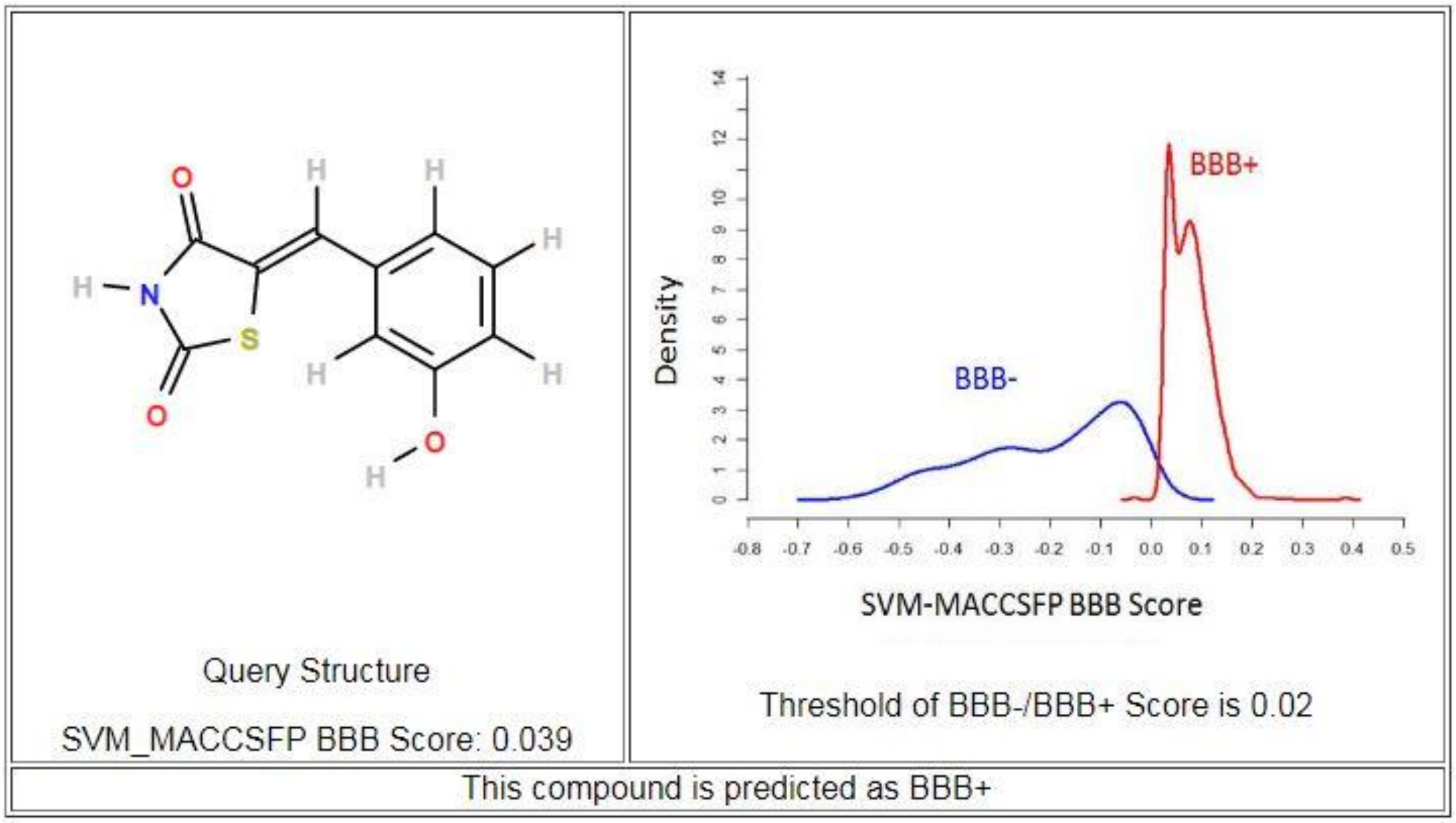

Figure S18. Diagram of the capability of the CHT3 in passing from BBB. The passage of compound CHT3 was predicted through the blood-brain barrier using the BBB Predictor server.

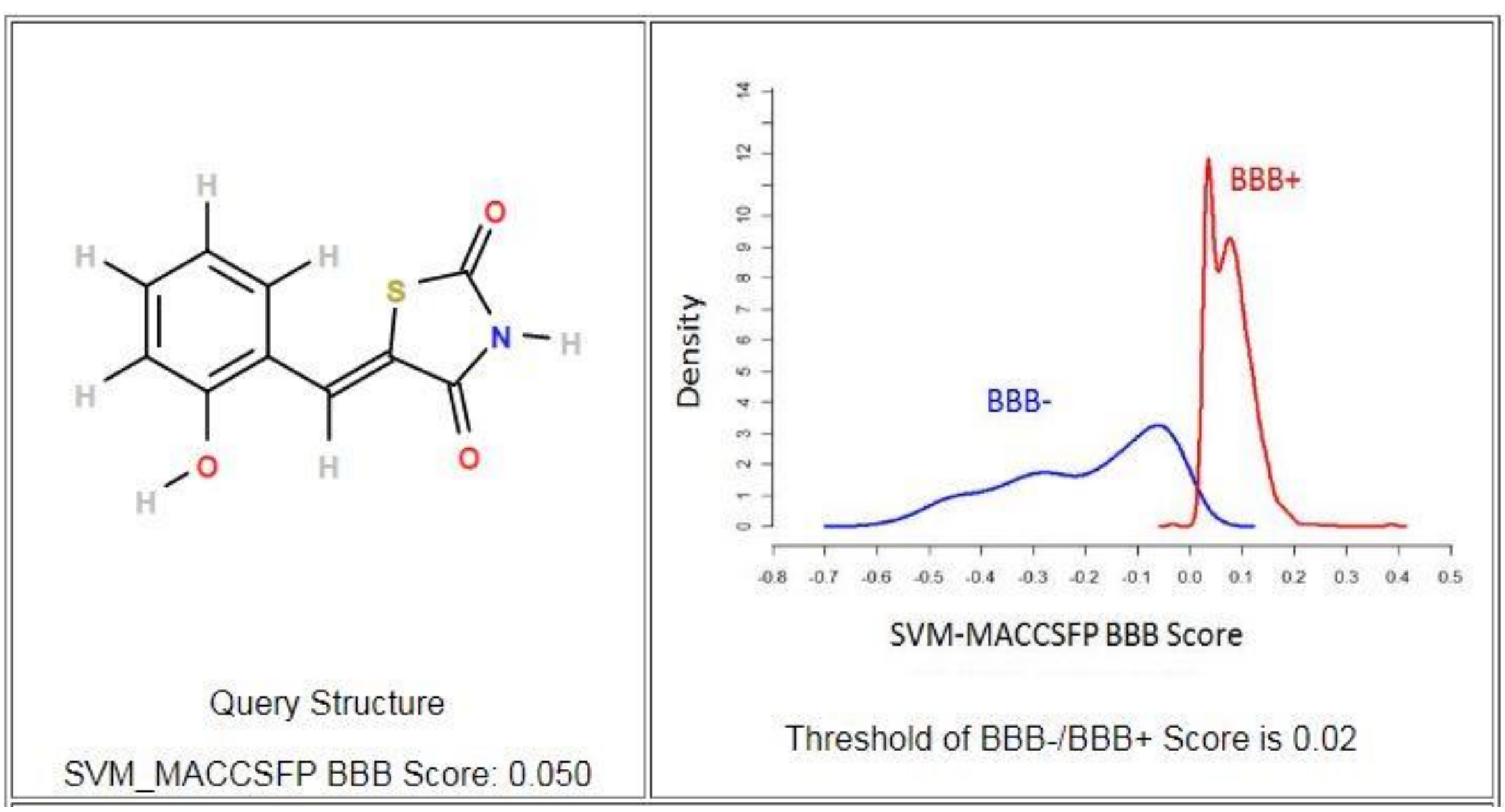

Figure S19. Diagram of the capability of the CHT4 in passing from BBB. The passage of compound CHT4 was predicted through the blood-brain barrier using the BBB Predictor server.

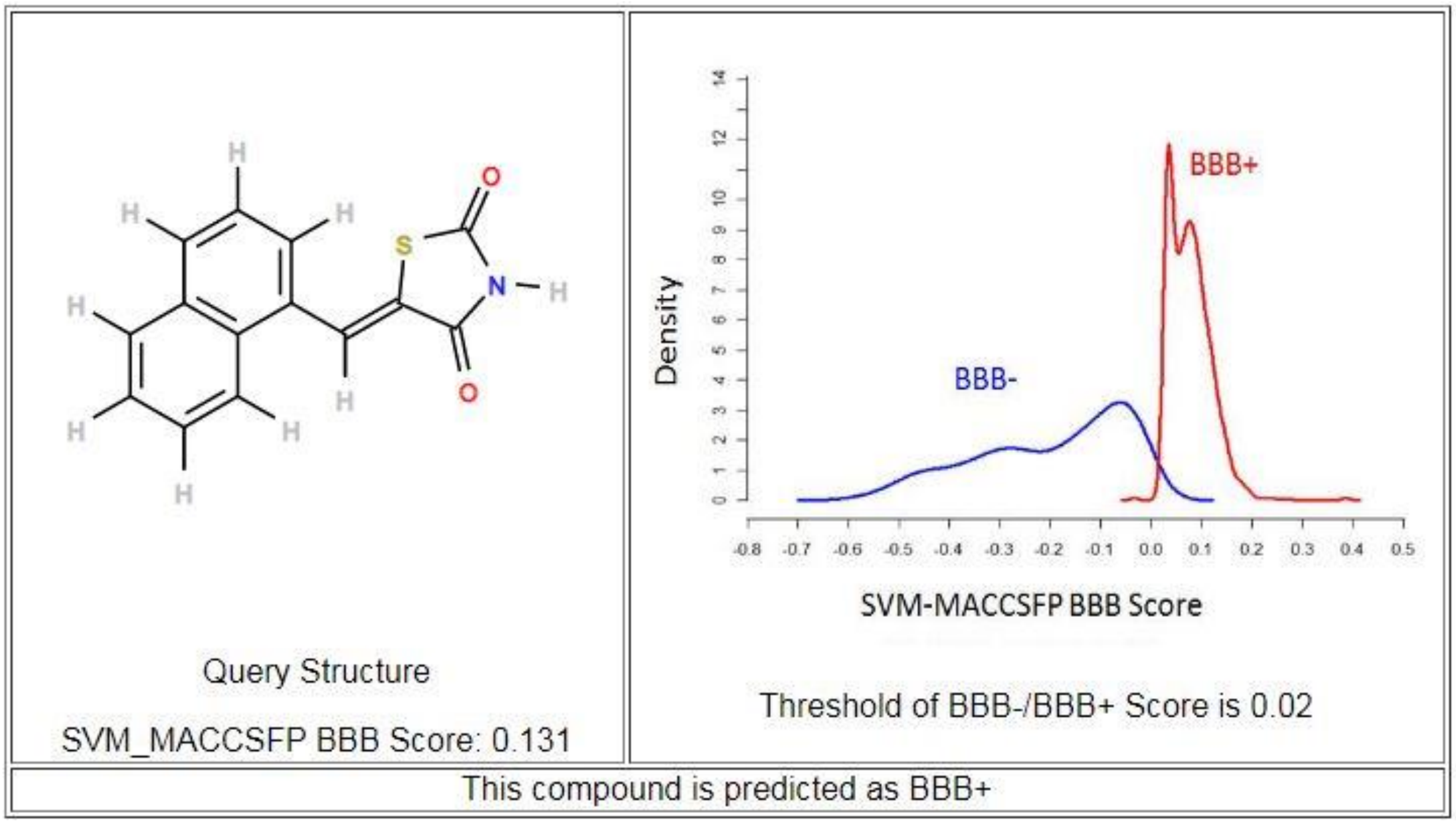

Figure S20. Diagram of the capability of the CHT5 in passing from BBB. The passage of compound CHT5 was predicted through the blood-brain barrier using the BBB Predictor server.

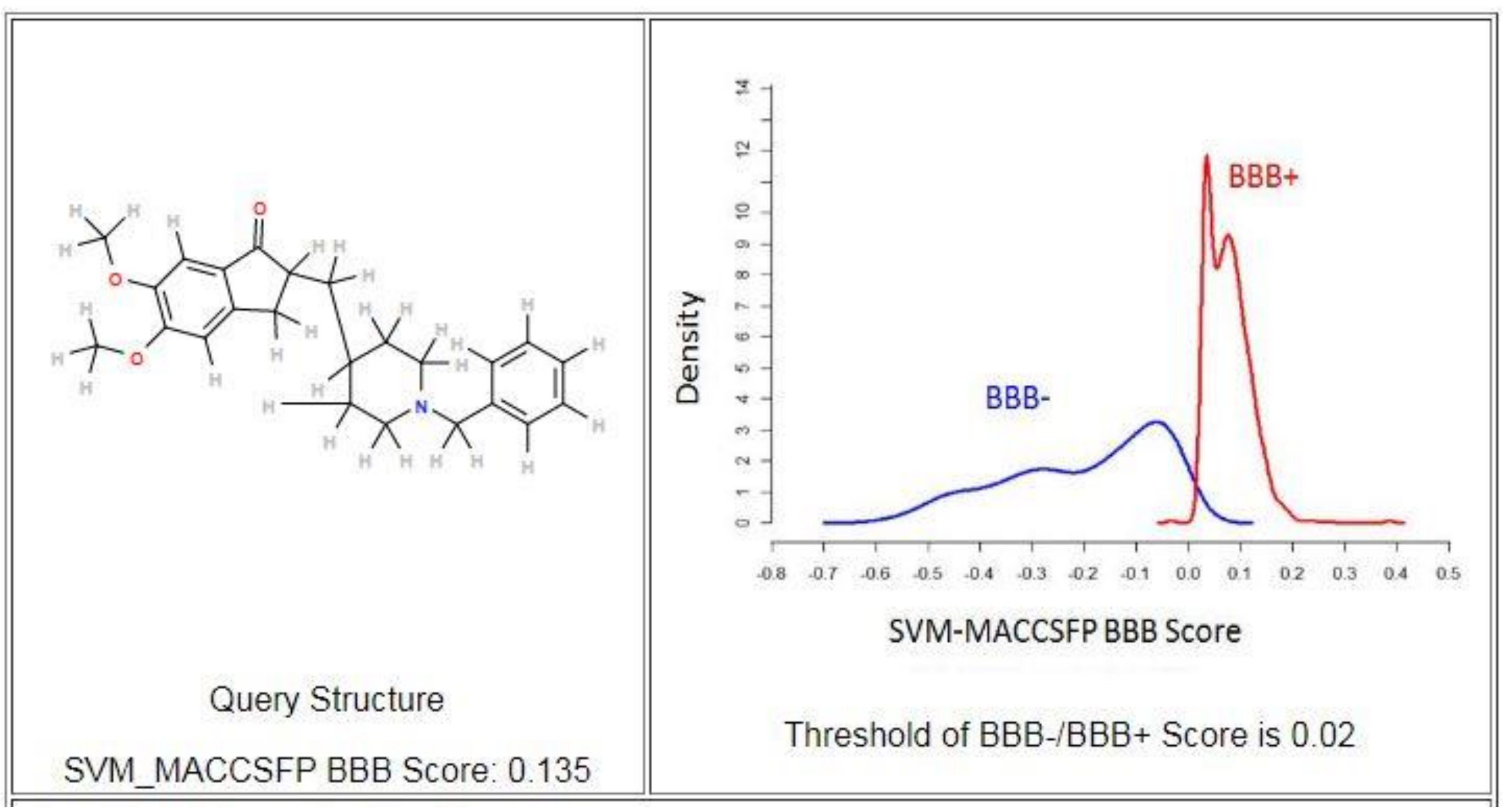

Figure S21. Diagram of the capability of the donepezil in passing from BBB. The passage of compound donepezil was predicted through the blood-brain barrier using the BBB Predictor server.

Original photos of Western blot gel

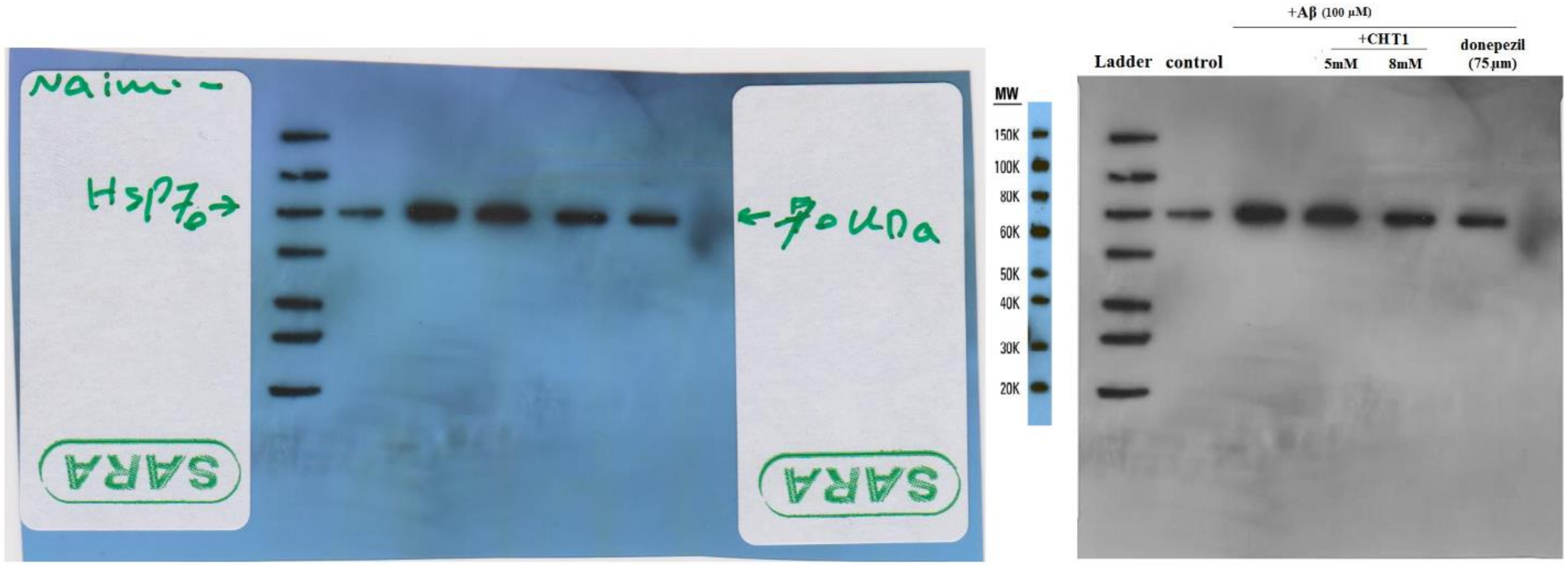

Figure S22. Original photo of western blot gel of HSP70. Representative western blot analysis of HSP70 in the PC12 cells pretreated with CHT1 (5 and 8 µm) followed by exposure to Aβ1-42 (100um). Donepezil is utilized as a positive control.

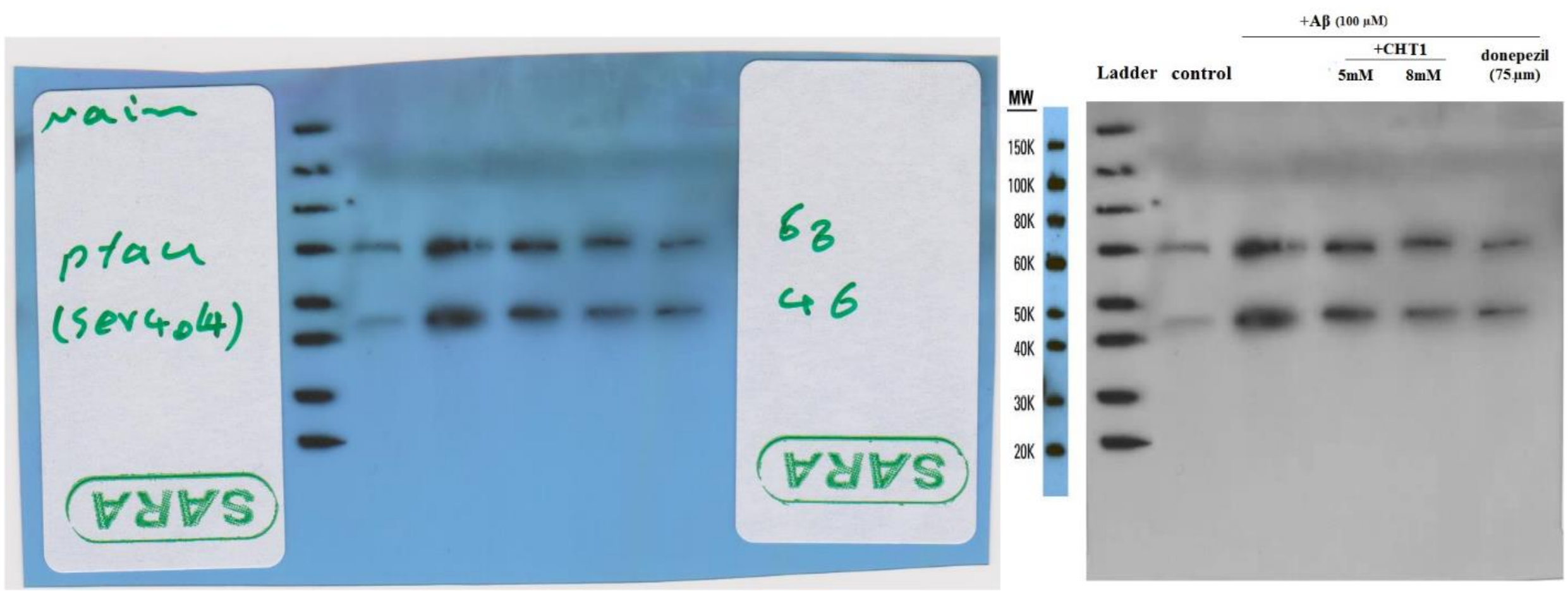

Figure S23. Original photo of western blot gel of p-Tau and Tau. Representative western blot analysis of p-Tau (68) and Tau (46 kDa) in the PC12 cells pretreated with CHT1 (5 and 8 µm) followed by exposure to Aβ1-42 (100um). Donepezil is utilized as a positive control.

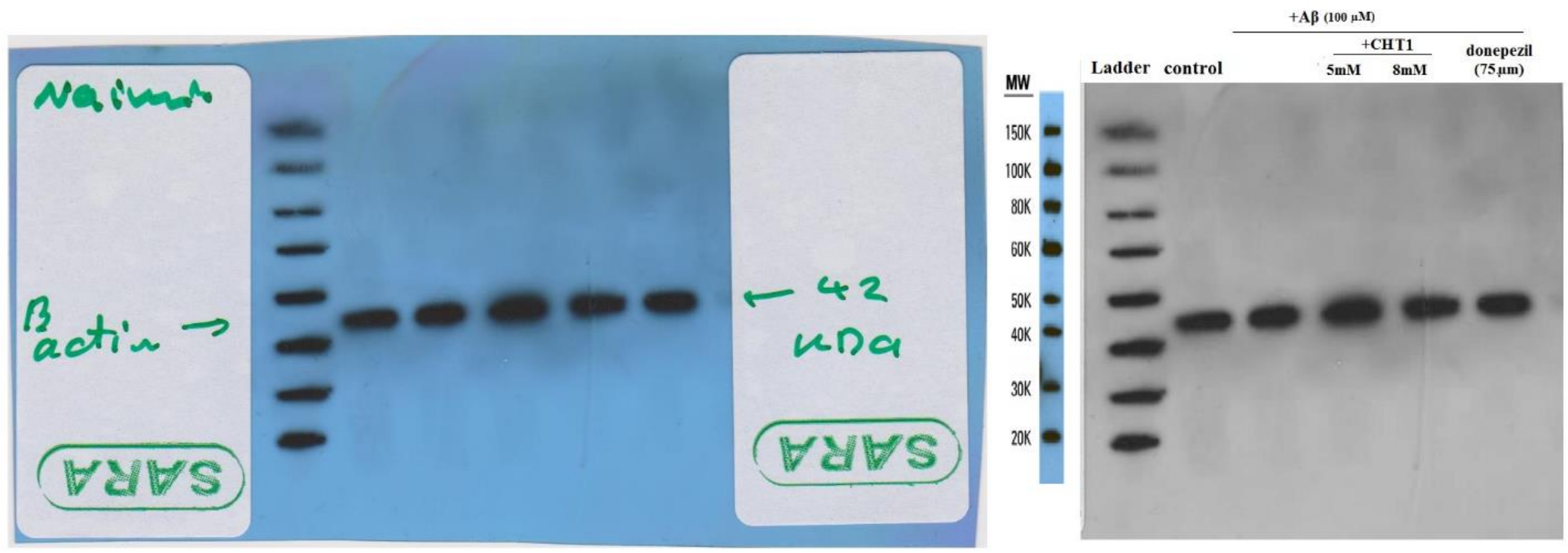

Figure S24. Original photo of western blot gel of β-actin. Representative western blot analysis of β-actin in the PC12 cells pretreated with CHT1 (5 and 8 μm) followed by exposure to Aβ1-42 (100um). Donepezil is utilized as a positive control.
